# Supplementary material for: Long-read RNA sequencing identifies non-coding isoform switching as a regulator of cell fate
Source: Nat Commun. 2026 Sep 24;17:11111. doi: 10.1038/s41467-026-78011-z (PMC13696733; doi:10.1038/s41467-026-78011-z)
Supplement: Supplementary file 1 [file 41467_2026_78011_MOESM16_ESM.pdf]

nature portfolio

Corresponding author(s): Samer M.I. Hussein

Last updated by author(s): Mar 25, 2026

Reporting Summary

Nature Portfolio wishes to improve the reproducibility of the work that we publish. This form provides structure for consistency and transparency in reporting. For further information on Nature Portfolio policies, see our Editorial Policies and the Editorial Policy Checklist.

Statistics

For all statistical analyses, confirm that the following items are present in the figure legend, table legend, main text, or Methods section.

n/a	Confirmed	
☐	☒	The exact sample size (n) for each experimental group/condition, given as a discrete number and unit of measurement
☐	☒	A statement on whether measurements were taken from distinct samples or whether the same sample was measured repeatedly
☐	☒	The statistical test(s) used AND whether they are one- or two-sided<br>*Only common tests should be described solely by name; describe more complex techniques in the Methods section.*
☒	☐	A description of all covariates tested
☐	☒	A description of any assumptions or corrections, such as tests of normality and adjustment for multiple comparisons
☐	☒	A full description of the statistical parameters including central tendency (e.g. means) or other basic estimates (e.g. regression coefficient) AND variation (e.g. standard deviation) or associated estimates of uncertainty (e.g. confidence intervals)
☐	☒	For null hypothesis testing, the test statistic (e.g. F, t, r) with confidence intervals, effect sizes, degrees of freedom and P value noted<br>*Give P values as exact values whenever suitable.*
☒	☐	For Bayesian analysis, information on the choice of priors and Markov chain Monte Carlo settings
☒	☐	For hierarchical and complex designs, identification of the appropriate level for tests and full reporting of outcomes
☒	☐	Estimates of effect sizes (e.g. Cohen's d, Pearson's r), indicating how they were calculated

Our web collection on statistics for biologists contains articles on many of the points above.

Software and code

Policy information about availability of computer code

Data collection	Raw pod5 files from Oxford Nanopore technologies long read cDNA sequencing were basecalled using Guppy (v6.5.7) using super accurate settings. Barcodes were detected and reads were demultiplexed with the guppy_barcoder command. The resulting FASTQ reads were aligned to the GRCm38/mm10 mouse reference genome with Minimap2 using the following parameters: -aLx splice –cs = long.
Data analysis	Raw read counts were obtained with the featureCounts tool from the Subread package (v2.0.3), using the exon counting mode. EdgeR R-package (v3.42.4) was then used to normalize the data, calculate RNA abundance at the gene level (as counts per million reads (CPM)), and perform differential expression analyses (fold change ≥ 2 (up-regulation) or ≤ 0.5 (down-regulation) and FDR ≤ 0.05). GO term enrichment was assessed using the Database for Annotation, Visualization and Integrated Discovery (DAVID) (v2022q3) (Sherman et al., NAR 2022). Biological Processes (BP) annotations were analyzed with a cut-off of FDR ≤ 0.1 for significant enrichment. Hierarchical clustering analysis of whole transcriptome was performed using the hclust method in the cluster R package. To assess isoform expression, reads were oriented according to transcript proper strand and transcriptome assembly was then performed using the TAMA software. Isoforms from the resulting GTF files were classified using the SQANTI3 software, using the GRCm38/mm10 mouse transcriptome as reference. Isoforms without detected poly(A) motifs or showing signs of RT-switching events were removed. Differential alternative splicing analysis was perfomed with the SUPPA 2 software. The coding potential score of detected transcripts was assessed with the Coding-Potential Assessment Tool (CPAT) (v3.0.5). Differential isoform usage was assessed using the IsoQuant software (v3.9). The EdgeR R-package (v3.42.4) was then used to normalize the data, calculate transcripts abundance (as TPM), and perform differential expression analyses (fold change ≥ 2 (up-regulation) or ≤ 0.5 (down-regulation) and p-value ≤ 0.05).

For manuscripts utilizing custom algorithms or software that are central to the research but not yet described in published literature, software must be made available to editors and reviewers. We strongly encourage code deposition in a community repository (e.g. GitHub). See the Nature Portfolio guidelines for submitting code & software for further information.

Data

Policy information about availability of data

All manuscripts must include a data availability statement. This statement should provide the following information, where applicable:

- Accession codes, unique identifiers, or web links for publicly available datasets
- A description of any restrictions on data availability
- For clinical datasets or third party data, please ensure that the statement adheres to our policy

Source data are provided with this paper. Datasets from other referenced works can be accessed either in the NCBI Gene Expression Omnibus (GEO) repository using their GSE numbers or in the database of the EMBL's European Bioinformatics Institute using their E-MTAB numbers (all reference datasets and processed datasets with links and descriptions are summarized in Supplementary Data 3, 6 and 7). Long-read RNA sequencing raw data, filtered aligned reads and processed data, including mouse transcriptome annotation and expression level matrices, from this study are publicly available and have been deposited in the GEO database under the accession codes: GSE282319 (mouse reprogramming data, https://www.ncbi.nlm.nih.gov/geo/query/acc.cgi?acc=GSE282319) and GSE317557 (human knock-down data, https://www.ncbi.nlm.nih.gov/geo/query/acc.cgi?acc=GSE317557). All mass spectrometry files used in this study were deposited in MassIVE (http://massive.ucsd.edu) and assigned the identifier MSV000100321 (https://massive.ucsd.edu/ProteoSAFe/dataset.jsp?task=94497fd1dc824ed0a471a6638f159439). All cell lines and biological materials generated in this study are available from the corresponding author upon direct request.

Research involving human participants, their data, or biological material

Policy information about studies with human participants or human data. See also policy information about sex, gender (identity/presentation), and sexual orientation and race, ethnicity and racism.

Reporting on sex and gender	This study focuses on better understanding the process of mouse reprogramming through long-read RNA sequencing. We used male mouse lines for reprogramming, which highlighted a long non-coding RNA involved in reprogramming and pluripotent stem cells. We then validated the importance of this lncRNA in a human female embryonic stem cell line.
Reporting on race, ethnicity, or other socially relevant groupings	This study concerns the cellular mechanism of somatic cell reprogramming to induced pluripotency. Therefore, questions of social categorizations are not relevant to the current study.
Population characteristics	No human research participants were included in this study.
Recruitment	No human research participants were recruited for this study.
Ethics oversight	All human embryonic stem cell research in this study was conducted under the ethical approval of the Stem Cell Oversight Committee from the Canadian Institutes of Health Research (CIHR).

Note that full information on the approval of the study protocol must also be provided in the manuscript.

Field-specific reporting

Please select the one below that is the best fit for your research. If you are not sure, read the appropriate sections before making your selection.

☒ Life sciences ☐ Behavioural & social sciences ☐ Ecological, evolutionary & environmental sciences

For a reference copy of the document with all sections, see nature.com/documents/nr-reporting-summary-flat.pdf

Life sciences study design

All studies must disclose on these points even when the disclosure is negative.

Sample size	No predetermination of required sample size was performed.
Data exclusions	Low quality reads were excluded from our analysis. Additionally, after transcriptome assembly, isoforms with hallmarks of artifactual isoform identification (i.e. no detected poly(A), probable RT-switching event, and low expression isoforms) were filtered out.
Replication	We investigated isoform diversity in the context of reprogramming. We used two independent reprogramming systems (OKMS and OKS) to account for system-specific effects. To validate our findings, we investigated Snhg26, a gene undergoing isoform-switching in both systems, and found that it plays a role in reprogramming and pluripotency maintenance. These results were reproducible in different reprogramming systems and in human cells, where SNHG26 is also implicated in pluripotency maintenance.
Randomization	Randomization and control of covariates is not relevant to our study, due to the nature of a time-course study of somatic cell reprogramming.
Blinding	Blinding was not relevant for our study as knowing the identity of our reprogramming samples is essential for a time-course analysis.

Reporting for specific materials, systems and methods

We require information from authors about some types of materials, experimental systems and methods used in many studies. Here, indicate whether each material, system or method listed is relevant to your study. If you are not sure if a list item applies to your research, read the appropriate section before selecting a response.

Materials & experimental systems

n/a	Involved in the study
☐	☒ Antibodies
☐	☒ Eukaryotic cell lines
☒	☐ Palaeontology and archaeology
☐	☒ Animals and other organisms
☒	☐ Clinical data
☒	☐ Dual use research of concern
☒	☐ Plants

Methods

n/a	Involved in the study
☒	☐ ChIP-seq
☐	☒ Flow cytometry
☒	☐ MRI-based neuroimaging

Antibodies

Antibodies used	SOX2 (Cell Signaling Technology #23064 1/100) and GATA6 (Novus Biologicals #AF1700 1/80) ; Alexa Fluor 488 anti-goat (ThermoFisher Scientific #A21467 1/500) and 594 anti-rabbit (ThermoFisher Scientific #A21207 1/500) ; NANOG (R&D Systems #AF1997), OCT4 (Santa Cruz Biotechnology #sc-9081), and SOX2 (R&D Systems #MAB2018)
Validation	Antibodies were validated by the compagnies selling them.

Eukaryotic cell lines

Policy information about cell lines and Sex and Gender in Research

Cell line source(s)	Mouse embryonic stem cell lines, including R1 (RRID: CVCL_2167) and Oct4-GFP (R1 ESCs harboring a GFP reporter gene under the control of the Oct4 gene promoter from Viswanathan et al., Biotechnology and Bioengineering 2003) and human embryonic stem cell line H9 (RRID: CVCL_9773) were all gifted by Dr Andras Nagy, University of Toronto. BJ fibroblasts were purchased at ATCC (#CRL-2522).
Authentication	R1 and Oct4 GFP were validated to be mouse male pluripotent stem cell lines by long-read RNA sequencing (GEO : GSE282319) and RT-qPCRs (data available upon request). The presence of a pluripotency-dependent GFP transgene in the Oct4-GFP live was validated by fluorescence microscopy and flow cytometry. In all transgenic lines generated for this study, expression of the correct transgene was validated by long-read RNA sequencing (GEO : GSE282319). The H9 human embryonic stem cell (hESC) line was validated as a female hESC line by long-read RNA sequencing (data available upon request). BJ fibroblasts are certified by ATCC (#CRL-2522).
Mycoplasma contamination	All cell lines were tested and negative for mycoplasma.
Commonly misidentified lines (See ICLAC register)	*Name any commonly misidentified cell lines used in the study and provide a rationale for their use.*

Animals and other research organisms

Policy information about studies involving animals; ARRIVE guidelines recommended for reporting animal research, and Sex and Gender in Research

Laboratory animals	Pluripotency of the iPSCs generated in this study was validated either by chimera formation assay (using Ai14 strain mice (RRID: IMSR_JAX:007914) and CD-1 mice (Charles River)), as well as by teratoma formation assay (using the 129S1/SvImJ strain (The Jackson laboratory #002448)).
Wild animals	*Provide details on animals observed in or captured in the field; report species and age where possible. Describe how animals were caught and transported and what happened to captive animals after the study (if killed, explain why and describe method; if released, say where and when) OR state that the study did not involve wild animals.*
Reporting on sex	We assessed the ability of the induced pluripotent stem cells (iPSCs) generated in this study to contribute to the formation of chimera when aggregated with 8-cell morula embryos. The blastocysts were transferred to the uteri of pseudo pregnant females. Those chimeras were both male and female. The ability of the iPSCs to form teratomas was tested by injection into male mice.
Field-collected samples	*For laboratory work with field-collected samples, describe all relevant parameters such as housing, maintenance, temperature, photoperiod and end-of-experiment protocol OR state that the study did not involve samples collected from the field.*
Ethics oversight	All mouse breeding and experimentation followed protocols approved by the Research Ethics Board Office from McGill University

Ethics oversight

(protocol 07-37) Guideline for Breeding and Weaning and according to the regulations issued by the Committee for Animal Rights Protection of the State of Hessen (Regierungspraesidium Darmstadt). Procedures involving animals performed by TCP were in compliance with the Animals for Research Act of Ontario and the Guidelines of the Canadian Council on Animal Care. Animal Care Committee reviewed and approved all procedures conducted on animals at TCP.

Note that full information on the approval of the study protocol must also be provided in the manuscript.

Plants

Seed stocks

Report on the source of all seed stocks or other plant material used. If applicable, state the seed stock centre and catalogue number. If plant specimens were collected from the field, describe the collection location, date and sampling procedures.

Novel plant genotypes

Describe the methods by which all novel plant genotypes were produced. This includes those generated by transgenic approaches, gene editing, chemical/radiation-based mutagenesis and hybridization. For transgenic lines, describe the transformation method, the number of independent lines analyzed and the generation upon which experiments were performed. For gene-edited lines, describe the editor used, the endogenous sequence targeted for editing, the targeting guide RNA sequence (if applicable) and how the editor was applied.

Authentication

Describe any authentication procedures for each seed stock used or novel genotype generated. Describe any experiments used to assess the effect of a mutation and, where applicable, how potential secondary effects (e.g. second site T-DNA insertions, mosiacism, off-target gene editing) were examined.

Flow Cytometry

Plots

Confirm that:

☒ The axis labels state the marker and fluorochrome used (e.g. CD4-FITC).

☒ The axis scales are clearly visible. Include numbers along axes only for bottom left plot of group (a 'group' is an analysis of identical markers).

☒ All plots are contour plots with outliers or pseudocolor plots.

☒ A numerical value for number of cells or percentage (with statistics) is provided.

Methodology

Sample preparation

Flow cytometry was used to study mouse cells during the timeline of reprogramming, as well as for doubling-time assays. Cells were harvested using TrypLE™ Express Enzyme (ThermoFisher Scientific #12604021) for dissociation, washed with DPBS (ThermoFisher Scientific #14190250), resuspended in 500µL of DPBS and filtered through a 35µm cell strainer (Fisher Scientific #08-771-23).

Instrument

BD Accuri™ C6 Plus flow cytometer (BD Biosciences)

Software

Flow cytometry data were collected with the C6 Plus analysis software and analyzed with FlowJo (v10.8.1).

Cell population abundance

No sorting was performed.

Gating strategy

Cell debris were gated apart using an FSC-A x SSC-A plot. Then, singlets were gated using an FSC-A x FSC-H plot and the gating for fluorescent reporter-positive cells was determined using a reporter-negative control sample as well as separated samples each positive for the different reporter genes.

☒ Tick this box to confirm that a figure exemplifying the gating strategy is provided in the Supplementary Information.
