# Supplementary material for: Long-read RNA sequencing identifies non-coding isoform switching as a regulator of cell fate
Source: Nat Commun. 2026 Sep 24;17:11111. doi: 10.1038/s41467-026-78011-z (PMC13696733; doi:10.1038/s41467-026-78011-z)
Supplement: Supplementary file 2 [file 41467_2026_78011_MOESM17_ESM.pdf]

nature portfolio

# Peer Review File

# Long-read RNA sequencing identifies non-coding isoform switching as a regulator of cell fate

Corresponding Author: Dr Samer Hussein

**This file contains all reviewer reports in order by version, followed by all author rebuttals in order by version.**

Version 0:

Reviewer comments:

Reviewer #1

(Remarks to the Author)
Fort et al report a detailed analysis of transcriptome changes occurring during cell reprogramming in mouse using long-read RNA sequencing. Their results reveal extensive switches in alternative splicing, often following a biphasic pattern, including long non-coding RNAs. Manipulation of one lncRNA, Snhg26, either by overexpression or depletion, modulates the efficiency of cell reprogramming in both mouse and human.

These are interesting results that can significantly expand over previous studies the diversity of transcripts generated during reprogramming, including isoform switches affecting long non-coding transcripts. The use of long read RNA-seq is also a valuable addition over previous transcriptome studies of the process. The authors also provide proof-of-principle that one lncRNA not previously implicated in cell reprogramming, plays a functional role in this process.

In my opinion the following revisions would improve the manuscript:

1. The description of the reprogramming system in section 1 is confusing and should be explained in more detail for non-experts. For example, on one hand, DOX-inducible reprogramming factor transgenes are used, on the other DOX is removed to convert cells to iPSCs…; and this happens by "naturally silencing" of OKMS transgenes, but then a GFP reported under Oct4 gene promoter is activated…
2. At the end of section 2 it would be helpful to briefly summarise what aspects of the new analyses confirm/expand previous observations of transcriptome diversity in reprogramming systems and which observations are newly reported in this manuscript.
3. It is essential that the authors provide extensive orthologous validation of the isoform quantifications predicted by RNA-seq analyses. The still relatively low throughput of ONT sequencing can limit the precise quantification of alternative mRNA isoforms and therefore it is in the authors' best interest to show the extent to which isoform switches of different magnitudes, and affecting genes of different overall expression levels, can be validated by independent methods such as RT-PCR. It would be particularly valuable to have validation of switches affecting snhg26 lncRNA isoforms.
4. Figure 4A: are the numbers referring to the exact same alternative splicing events or to the genes undergoing alternative splicing during reprogramming? This should be clarified.
5. Figures 4E-F: can the predicted functional effects of FYN splicing switch be validated in the transcriptome datasets (e.g. changes in expression of downstream targets of FYN)?
6. Figure 5A (and others): it would be good to clarify what upregulation or downregulation of isoforms means. For example, for cassette exons, increases in exon inclusion will be accompanied by concomitant decreases in exon skipping and vice versa. How are these two events counted in these graphs? The same for Alt 5' or 3' ss or intron retention events.
7. Figure 5E: similar to point 5 above, can the authors validate downstream effects of changes in SRSF2/SRSF3 levels by looking at changes in alternative splicing of known target genes of these factors?
8. Page 13: I am not sure I follow the argument that because "there is a negative selection against lncRNAs to undergo IS", those "lncRNAs that undergo IS may harbor important functions". The negative selection may not be as strong as not to allow some lncRNAs to undergo IS switches without necessarily implying important functional roles for the switch.
9. Figures 6A-B: the selection of the 19 candidates (p-13, line 31) should be better explained and justified in the text. Panel B: the 2 lncRNAs selected were the only ones expressed in iPSC cells or (p.14, line 1) the ones particularly highly expressed? How was this threshold established?
10. Figure 7: the exact snhg26 isoform used in the overexpression experiments and its choice given the IS described in

Figure 6 should be clearly explained and justified.
11. Given the relevance to prove effective reprogramming, I would recommend to bring the embryo data now shown in Extended Data of Figure 8H to the main Figure 7.
12. Figure 8F: it would be good to include also quantification data of colony sizes in addition to colony numbers.

Juan Valcarcel

Reviewer #2

(Remarks to the Author)
Authors hereby present a very interesting and sound model for cellular reprogramming, and generate a valuable lrRNA-seq dataset covering the process across different timepoints. Alternative isoform expression is a key regulatory mechanism for many biological processes, including differentiation, but it is often overlooked or only analyzed from an exon-centric perspective. In this context, this research is both timely and relevant.

Therefore, my comments do not intend to put the biological insights obtained from the study into question, but improve the decisions made during long-read data processing and transcriptome reconstruction. The usage of SQANTI3 for transcriptome curation in this study shows that authors are aware that lrRNAseq data is rich in artifacts. However, more extensive analyses and filtering should be performed before trusting the data, especially regarding novel isoforms. I trust that these suggestions will help authors improve their manuscript and contribute to enhance the relevance and soundness of their discoveries.

Main comments:

1. The transcriptome presented in the manuscript is very large, and high percentage of novel isoforms is concerning, particularly when it comes to NNC transcripts. I suggest the following measures are taken to ensure that the isoforms included are realiable and provide readers with a detailed account of the transcriptome's properties:

1.A. Even though polyA motif and TSS (i.e. CAGE data) data are incorporated, it is important to look at quality metrics that are specific to the transcript area presenting novelty (in this case, the novel splice junctions). This could be any feature in SQANTI3 related to junction coverage by short reads, FL counts or even expression. Further evidence needs to be supplied to validate these novel transcripts, filtering those cases where anomalies are found (i.e. novel junctions with no coverage). Validation with short reads would be ideal and, if no compatible short read dataset is available, a transcript expression threshold should be applied within the rules filter to ensure that the novel isoforms are reliable. Detection of the junctions in a minimum percentage of the replicates would be another option to validate these potentially novel isoforms.

1.B. Related to the previous point, it would be interesting to see the distribution of some relevant metrics across categories (if available), such as expression/FL count, minimum coverage (if short reads are available), length/exon number, CAGE peak and polyA motif detection, distance to TSS/TTS, etc. to better understand whether these transcripts are generated by some kind of library-related artifact, and make informed decisions to remove them. This detailed description of the properties of transcripts and the category they belong to would fit nicely as a supplementary figure, including information before and after filtering to ensure that the artifacts have been successfully removed.

2. Regarding filtering, I have several additional concerns:

2.A. Authors should pay attention to the default rules filter in SQANTI3, and note that it does not consider polyA motif or CAGE peak information. Per the author's description, I understand that isoforms not including a polyA motif or exhibiting RT switching are being eliminated after the rescue is run. RT switching-based filtering is already incorporated in the default rules, but it would be best to include polyA motif detection as part of the rules filter and for all isoform categories, as the removal of these transcripts prior to the rescue will surely affect how this step is performed, impacting the final composition of the transcriptome.

2.B. In addition, it seems that no filtering decision is made base on CAGE peak information, in spite of using this information when running SQANTI3 QC. It would be adviseable to add this to the rules filter (via the within CAGE peak metric or using threshold of distance to the nearest CAGE peak) for all categories. Also, the details of the CAGE peak dataset being used for this purpose is not included in the relevant methods section.

2.C. I suggest completing polyA and CAGE peak filtering with other annotation and data-based TSS and TTS quality metrics, such as the distance to the annotated TSS/TTS and the TSS ratio, as it has been shown to be relevant to remove artifacts, especially within the ISM category.

Authors are referred to the SQANTI3 manuscript for ideas as to how these filtering criteria can be incorporated into a custom-made rules filter script.

3. Regarding the results oultined in section 3 (i.e. Exclusive regulation of non-coding isoforms):

3.A. I am curious as to why authors do not start by performing an analysis of coding vs non-coding isoforms as a whole. It might be that the results described here can be attributed to the properties of non-coding isoforms as a whole, not solely to novel non-coding isoforms. In other words, do coding vs non-coding isoform results replicate those observed for non-coding

NNC?

3.B. It is not clear from the methods whether authors derived these results by performing multiple pairwise analyses between D0 and the different phases with edgeR. If so, the correct approach to this analysis would be to fit one GLM model per isoform using edgeR with the different phases as a covariate, deriving significant changes in isoform expression across reprogramming (using the adjusted p-value), and then test each of the coefficients for significant changes in expression. I apologize if this was the approach used in the first place, and would therefore recommend authors to include a more detailed account of this step in the relevant methods section and make the code available to ensure reproducibility and avoid confusion. In addition, I believe puting the differential gene expression and differential isoform expression analysis sections in the methods next to each other, and after the transcriptome assembly step, would help follow the text.

4. I am aware that the implementation of these comments may alter some of the transcriptome-wide conclusions in the subsequent sections of the manuscript. I wish to thank the authors in advance for their time and dedication, and look forward to reading the revised version of their work.

Minor coments:
- Page 6, line 22-23: “Novel In Catalog (NIC)” instead of “Not In Catalog”

Reviewer #3

(Remarks to the Author)

This study aims to identify novel functional isoforms regulating cell fate using long-read RNA sequencing (lrRNAseq). The authors employ two reprogramming systems, OKMS and OKS, to distinguish c-Myc-dependent and -independent effects on alternative splicing (AS) and isoform regulation. They identify multiple previously undetected isoforms with dynamic expression patterns during reprogramming. Notably, non-coding isoforms are negatively selected during mid-phase reprogramming, while long non-coding RNAs (lncRNAs) undergoing IS appear functionally important for pluripotency acquisition. Finally, the study demonstrates that overexpression of the lncRNA Snhg26 enhances reprogramming efficiency. While the study comprehensively profiles long-read RNA seq during reprogramming, the manuscript is largely descriptive and functional validations requires a careful re-examination the use of controls and targeting strategies.
Major Comments
1. The study includes 22x R9 and 13x R10 samples. The differences in read accuracy or coverage can significantly lead to biases isoform detection. Please explain the extent of batch effects observed across the samples and how the batch effects across these sequencing versions were accounted for.
2. D14 and D20 OKS samples are grouped as "late phase," yet Extended Data Figure 2C shows substantial differential gene expression between them. Please clarify this point, including ONT R10 data for whole-transcriptome DE and clustering analysis (OKMS vs. OKS) could refine phase definitions and further strengthen conclusions.
3. Somatic reprogramming process is highly heterogeneous – even with Dox system (https://www.cell.com/fulltext/S0092-86741201021-5). What is the level of heterogeneity in the dataset – such as running single cell RNA-seq- during the progression of OKMS and OKS reprogramming will reveal this and further clarify whether the isoforms are stage specific vs cell type/state specific.
4. (Figure 2A) How does the percentage distribution of isoform categories (e.g., NNC, annotated) vary across reprogramming stages? Are certain categories underrepresented due to read coverage biases? Providing read counts per category would address detection sensitivity.
5. (Section 3, Line 27) How many "isoforms from novel genes" were analyzed? Are they exclusively non-coding, or do some retain coding potential?
6. Defining novel transcripts requires strong transcript evidence. Systematically annotate novel transcripts with well-annotated 5’ support such as FANTOM and use orthologous tools such as BAMBU (https://www.nature.com/articles/s41592-023-01908-w) to provide confidence to these novel transcripts as opposed to potential spurious or degraded transcripts. Moreover, majority of lncRNAs are non-poly-adenylated, yet many were discovered using poly-dT reverse transcription library prep in ONT. Please explain to what extend the newly discovered lncRNAs are polyA+ and polyA- and verify them.
7. Are there shared non-coding isoforms between OKMS and OKS (Figure 2F)? Identifying these could reveal c-Myc-independent regulatory mechanisms.
8. The authors use both ΔPSI and TPM-based DEIs to define Isoform Switching. Are the results consistent between methods? A direct comparison would validate robustness. What are the functional implications to make the manuscript less descriptive.
9. What is the link between AS events and NNC isoforms? Do NNC isoforms originate from genes with AS events? Overlap analysis (e.g., Venn diagrams) could clarify whether AS directly generates NNC transcripts.
10. (Section 5) How does exon skipping in MBNL2 correlate with its isoform expression? Is the loss of protein-coding function a direct IS effect or a secondary stress response? Controls (e.g., stress-induced splicing in non-reprogramming cells) would help establish causality.
11. In functional validation of Snhg26, is Snhg26 or its specific isoform (G38722.217) overexpressed? To test causality, perturbing splicing factors could directly determine whether IS drives reprogramming outcomes.
12. c-Myc-Independent Isoform Switching. Were common IS events between OKMS and OKS systems analyzed? This could reveal splicing regulation independent of c-Myc’s proliferative effects.
13. Targeting Snhg26 with CRISPRi prevents transcription at the promoter – thus not able to distinguish the role of transcript isoforms versus the gene. Since the author’s work stems from the fact that there are isoform specific effects during reprogramming, the authors should functionally validate these claims using a method that can target the isoform more

specifically. This is further noted using the ASO where the gene is suppressed as opposed to transcript specific effects. Please consider an experiment to selectively target one isoform and not the other to support the claim that isoform is causal to reprogramming rather than the gene itself. Further note that ASOs can interfere with nascent/primary transcript, thus they are not selective in suppressing specific isoforms. Splicing switching oligos or CRISPR designed to remove specific exons can clearly demonstrate the functional specificity of Snhg26 isoforms.

Minor Comments
1. CPAT Analysis Thresholds. The use of –min-orf 10 (default: 75) may inflate false-positive coding predictions. Justify this threshold or provide validation (e.g., ribosome profiling) to confirm coding potential.

I co-reviewed this manuscript with one of the reviewers who provided the listed reports. This is part of the Nature Communications initiative to facilitate training in peer review and to provide appropriate recognition for Early Career Researchers who co-review manuscripts.

Reviewer #4

(Remarks to the Author)
The reviewer has carefully evaluated the manuscript entitled "Long-read RNA sequencing identifies non-coding isoform switching as a regulator of cell fate" by Fort et al. This study employs long-read RNA sequencing (lrRNAseq) to analyze RNA isoform diversity during the reprogramming of mouse neural progenitor cells (NPCs) into induced pluripotent stem cells (iPSCs). A large number of novel splicing isoforms and isoform switching (IS) events were identified, and a novel long non-coding RNA (lncRNA), Snhg26, was found to promote reprogramming via isoform switching and maintain pluripotency in both mouse and human embryonic stem cells, thus revealing the key role of isoform diversity in cell fate regulation. Overall, this study pioneers the application of long-read RNA sequencing to dissect alternative splicing and isoform diversity during reprogramming, an innovative approach that provides a new perspective for understanding the reprogramming process. However, the reviewer requests that the authors address the following revisions before a final decision on publication is made.
MAJOR POINTS
1、The reprograming efficiency and the quality of the iPSC.
The reprogramming system employed in this manuscript exhibits low efficiency, which may compromise the assessment of the actual efficacy of the reprogramming process and, consequently, the validity of the main findings. It remains unclear whether the reported phenotypes would persist if a more efficient reprogramming system were adopted. Furthermore, rigorous functional validation of the resulting induced pluripotent stem cells—such as teratoma and chimera formation assays—is necessary to confirm the reliability of the reprogramming experiments.
2、The phenotypic and molecular mechanisms of snhg26 in somatic cell reprogramming.
The manuscript does not clearly define the key phenotype attributed to SNHG26 during reprogramming. The authors claim "Snhg26 lncRNA enhances the reprogramming route to pluripotency"，yet it remains ambiguous whether this reflects an increase in clonal reprogramming efficiency or an acceleration of reprogramming kinetics. More detailed analyses and rigorous demonstrations are required to resolve this distinction.
3、The role and molecular mechanism of snhg26 lncRNA in maintaining the pluripotency of embryonic stem cells.
Using a knock-down strategy, the authors examined the role of the lncRNA SNHG26 in maintaining pluripotency in both murine and human embryonic stem cells (ESCs). They report that SNHG26 lncRNA Knock down reduces clonal-formation efficiency. However, this observation alone does not establish SNHG26 lncRNA as an essential factor for pluripotency maintenance in ESCs. To substantiate such a claim, the authors must perform extended serial passaging and rigorously quantify core pluripotency markers as well as lineage-differentiation genes. Otherwise, the authors should turn down their claims. In addition, Knockout of the lncRNA shows no detectable impact on core pluripotency genes in human embryonic stem cells (hESCs), raising concerns about its functional relevance.

MINOR POINTS
4. In Figure 1, the design of an efficient reprogramming system is not the main research finding of this study. This section merely validates the feasibility of using long-read sequencing to detect gene expression during reprogramming.
5. In Figure 1, Why did the authors choose NPCs as the starting cell type for constructing the reprogramming system? In Figure 6, the expression pattern of lncRNA (2700038G22Rik) in the early stage of NPC reprogramming is significantly different from that in MEF and TTF reprogramming.
6. On page 4, lines 16–17, the statement "overactivation of c-Myc induces transcriptional and splicing events through its role in promoting proliferation" requires relevant references. Additionally, no corresponding results or explanations for this conclusion are provided in the subsequent analysis.
7. In Figure 4, common splicing phenotypes of Mbnl2 and Fyn were identified by comparison with other datasets, which supports the relevant conclusions. However, the highlight of this part is "the strength of using lrRNAseq to detect new isoforms and splicing events compared to other technologies." In comparison, the analysis of the function of the novel isoforms of the 7890 gene, which is specific to lrRNAseq, can better illustrate this point.
8. In Figure 5, it is mentioned that isoform variations of Srsf2/Srsf3 related to "mRNA processing and RNA splicing" are present. The manuscript only provides statistical and descriptive analysis of these different isoforms, and does not analyze their specific regulatory effects on RNA splicing or reprogramming. This seems to be more important.
9. On page 14, line 6, the meaning of "meeting all the above criteria without ambiguity" is not clear. Why does the author focus on 2700038g22Rik for further analysis. What is the result of 2010204k13Rik for further analysis.
10. In Figure 7 or extended data figure 7, the cell morphology and colony formation under the same visual field at different time points during OKS+ Snhg26 lncRNA/ OKS reprogramming should be provided to support the conclusion that Snhg26

promotes the increase of iPSC colony formation.
11. On page 17, line 4, as one of the most important findings in the manuscript, "cell intrinsic mechanisms" needs to be displayed and explained in the main figure or text.
12. In Figure 8C, the phenotype of Snhg26 knockdown is not significant. Furthermore, when analyzing the expression difference between SALL4 and REX1, the p value seems to be wrong. It would be better if the p value could be displayed in the graph.
13. In Extended Data Figure 9C, has the specificity of the Snhg26-specific probes been validated?
14. Some images lack scalebars, such as those in Figure 8F, Extended Data Figure 8H and Extended Data Figure 9G, H.
15. Marker selection. DPPA3 is a more robust and representative marker for human naïve pluripotency than DPPA4.

Reviewer #5

(Remarks to the Author)
I co-reviewed this manuscript with one of the reviewers who provided the listed reports. This is part of the Nature Communications initiative to facilitate training in peer review and to provide appropriate recognition for Early Career Researchers who co-review manuscripts.

Version 1:

Reviewer comments:

Reviewer #1

(Remarks to the Author)
The authors have adequately addressed the issues raised in my previous report and I am happy to recommend publication of the paper in Nature Communications.

Reviewer #2

(Remarks to the Author)
I wish to thank authors for their kind responses and for thoroughly answering the comments in my report and modifying their manuscript accordingly. My concerns have now been sufficiently addressed, and I believe this round of review has contributed to improve the quality of the manuscript.

However, I remain slightly unconvinced regarding the decision to include isoforms that lack validation in one or more of the structural sources of novelty (TSS, TTS or splice junction). The way the manuscript is currently presented, I think readers might wonder why these isoforms remain included, if sufficient validation could not be achieved. Perhaps the criteria behind this decision should be openly discussed. I understand that authors are concerned with being too stringent and perhaps removing some valuable signal from their data, but I would be cautious to point out that showing similar properties to those of fully validated isoforms is sufficient evidence to preserve them in the transcriptome.

To avoid raising doubts among readers, I suggest authors explicitly address this decision in the paper, whether it is in the corresponding section of the manuscript or later in the discussion. I do agree that using data sources and databases that do not exactly match the system in which the data was generated might limit the extent to which isoforms can be fully validated, and this could be commented as well.

I agree that it is reassuring, however, that all examples of biologically-relevant isoforms discussed later in the manuscript have multiple sources of evidence supporting them.

Finally, I wish to congratulate authors on their work and, especially, on their openness to criticism and willingness to incorporate the feedback from all four reviewers. It has definitely eased the review process and should not go unnoticed.

Reviewer #3

(Remarks to the Author)
We have no further comments to the authors

Reviewer #4

(Remarks to the Author)
The revised manuscript is substantially improved. The authors have adequately addressed several major concerns, including chimera/teratoma validation of Snhg26.217-derived iPSCs, mechanistic dissection of reprogramming efficiency vs. kinetics (limiting dilution assay, density-based analysis), and the compelling blastoid rescue experiment showing isoform-specific rescue by Snhg26.217 but not .216. However, the following issues must be resolved before acceptance:
Major comments:
1. Serial passaging after Snhg26 knockdown/KO in mouse ESCs is absent. Only 5 days of CRISPRi knockdown is shown. This is insufficient to claim a role in pluripotency maintenance. Authors must provide extended passaging (≥5 passages) with pluripotency marker quantification, or explicitly reframe the claim as "acute pluripotency perturbation" rather than

maintenance.
2. Teratoma formation for the baseline OKMS/OKS iPSC lines (Fig. 1) remains unshown. Please either add this data or justify why chimera formation alone suffices.
3. SNHG26 knockdown in hESCs still fails to significantly downregulate OCT4/NANOG. The authors' explanation (primed vs. naïve state) is reasonable but should be more prominently stated as a limitation, not a resolution. The 24–42% changes in secondary markers are modest and should not be overstated.
Minor comments:
4. Add 1–2 sentences in Results justifying the choice of NPCs as the starting cell type.
5. Display exact p-values directly on Supplementary Fig. 16b graph (SALL4, REX1 changes in hESCs).
6. Specify in Methods how Snhg26 ISH probe specificity was validated (e.g., knockdown control).
7. Verify scale bars are present in all image panels (Fig. 8F; Supplementary Figs. 13, 15).
8. For Fig. 5 (Srsf2/Srsf3 isoforms): add at least a brief overlap analysis with published SRSF2/SRSF3 splicing targets, or explicitly frame this section as hypothesis-generating.

**Open Access** This Peer Review File is licensed under a Creative Commons Attribution 4.0 International License, which permits use, sharing, adaptation, distribution and reproduction in any medium or format, as long as you give appropriate credit to the original author(s) and the source, provide a link to the Creative Commons license, and indicate if changes were made.
In cases where reviewers are anonymous, credit should be given to 'Anonymous Referee' and the source.
The images or other third party material in this Peer Review File are included in the article’s Creative Commons license, unless indicated otherwise in a credit line to the material. If material is not included in the article’s Creative Commons license and your intended use is not permitted by statutory regulation or exceeds the permitted use, you will need to obtain permission directly from the copyright holder.
To view a copy of this license, visit https://creativecommons.org/licenses/by/4.0/

**Response to referees**

We would like to first thank the editorial office and the reviewers for their thorough review of the manuscript and for providing constructive criticism and comments to improve it. Their comments helped us generate a much-improved revised manuscript. We are confident that we have reached the level that will satisfy both the reviewers and the editor of the journal. Please find below our point-by-point responses to the reviewers' comments. Also, our changes are highlighted in red in the manuscript text.

**Reviewer #1 (Remarks to the Author):**

> *Fort et al report a detailed analysis of transcriptome changes occurring during cell reprogramming in mouse using long-read RNA sequencing. Their results reveal extensive switches in alternative splicing, often following a biphasic pattern, including long non-coding RNAs. Manipulation of one lncRNA, Snhg26, either by overexpression or depletion, modulates the efficiency of cell reprogramming in both mouse and human.*
> *These are interesting results that can significantly expand over previous studies the diversity of transcripts generated during reprogramming, including isoform switches affecting long non-coding transcripts. The use of long read RNA-seq is also a valuable addition over previous transcriptome studies of the process. The authors also provide proof-of-principle that one lncRNA not previously implicated in cell reprogramming, plays a functional role in this process.*

We would like to thank the reviewer for the thorough review of the manuscript and for their input and time dedicated to reviewing our manuscript.

> *In my opinion the following revisions would improve the manuscript:*
>
> *1. The description of the reprogramming system in section 1 is confusing and should be explained in more detail for non-experts. For example, on one hand, DOX-inducible reprogramming factor transgenes are used, on the other DOX is removed to convert cells to iPSCs…; and this happens by "naturally silencing" of OKMS transgenes, but then a GFP reported under Oct4 gene promoter is activated…*

We thank the reviewer for this comment which raised an important issue about the clarity of our manuscript. We agree that the description of the reprogramming system may be difficult to follow and understand for non-experts. We had provided a detailed explanation of the reprogramming system in the Supplementary Note document; however, we recognize that most readers will not necessarily explore it. Therefore, we modified the description of the reprogramming system directly in section 1 ("Defining reprogramming stages by lrRNAseq") of the article. We hope that this will provide the necessary explanations for any reader.

*2. At the end of section 2 it would be helpful to briefly summarise what aspects of the new analyses confirm/expand previous observations of transcriptome diversity in reprogramming systems and which observations are newly reported in this manuscript.*

We thank the reviewer for this helpful suggestion. Although several studies done by our peers have analyzed the AS patterns at play during reprogramming, our manuscript is, to the best of our knowledge, the first to report deep analysis of transcriptome diversity, in any type of reprogramming system, since it is the only one using long-read RNAseq (lrRNAseq) and thus addressing isoform diversity. Therefore, we used our assembled transcriptome to compare and validate our findings in multiple other datasets, such as in new **Figures 4a**, **6b**, and **6h-j**, and **Supplementary Figures 9h**, **10a-c**, and **11b-f**. Our data also provides the classification of all the transcripts expressed during somatic cell reprogramming, as well as addresses their coding potential. Here, we demonstrate how changes between coding and non-coding isoforms of a gene play a major role in gene regulation during reprogramming. We show this using single gene examples (e.g. *Srsf2* in **Fig. 5i** and **Supplementary Fig. 9f-g**), as well as globally (such as in **Figures 4j-k** and **5g)**, and to our knowledge, this was never shown before because of lack of long-read sequencing. So as requested, we emphasized this notion in the introduction and at different parts of section 2 ("Unprecedented isoform diversity and tight regulation of non-coding isoforms during reprogramming") to clarify our findings.

*3. It is essential that the authors provide extensive orthologous validation of the isoform quantifications predicted by RNA-seq analyses. The still relatively low throughput of ONT sequencing can limit the precise quantification of alternative mRNA isoforms and therefore it is in the authors' best interest to show the extent to which isoform switches of different magnitudes, and affecting genes of different overall expression levels, can be validated by independent methods such as RT-PCR. It would be particularly valuable to have validation of switches affecting snhg26 lncRNA isoforms.*

The reviewer raises an important point that isoform quantifications can be challenging. For this reason, we added 10 more samples (a list of the 45 samples can be found in the "lrRNAseq-samples" tab in **Supplementary Table 1**) using R10 flow cells (i.e. better base calling and depth than the previous R9) and re-did all the quantifications using updated versions of quantification software, such as IsoQuant (v3.9.0), to more accurately quantify isoform changes. Moreover, our sequencing depth is higher than many long-read sequencing datasets that are currently published. We have an average of 4.8 million reads and 5.2 Gb per R10 sample (see "Statistics-lrRNAseq-reads" tab in **Supplementary Table 1**), compared to on average 1 million reads and 0.7 Gb per sample by other datasets such as Qiao *et al*. Nat. Comm. 2020, Zheng *et al*., Sci. Data 2021, Torre *et al*. Nat. Comm. 2023, Jones *et al*., Mol. Brain 2024, and Sun *et al*., Nat. Comm. 2021, i.e. a ~7-fold increase in total depth. Nonetheless, we did perform gene-specific RT-PCR validation of the *Snhg26* lncRNA isoform switching event detected during reprogramming. This data is now shown in **Supplementary Fig. 11a**. Here, we opted to use long-read DNA sequencing of the PCR amplicons to detect the changes and manually count the reads that correspond to each of the two *Snhg26* isoforms (*Snhg26.216* and *Snhg26.217*) at different phases of both OKMS and OKS reprogramming. We did not run PCRs on a gel because simply running a gel would be difficult to get a clear picture. For example, it is often either the PCR products were too close in size, or we

would need to rely on negative PCRs (meaning no PCR product = splicing event) to detect the splicing and isoform switch. This is also why it was difficult to demonstrate isoform switching by RT-PCR with other genes, such as *Srsfs*, *Fyn*, and *Mbnl2*. However, we did validate isoform expression changes for *Srsf2* and *Snhg26* using short-read RNAseq data from B cell reprogramming (Stadhouders *et al*., Nat. Genet. 2018), where we observed similar isoform expression patterns as shown in our data (**Supplementary Figures 9h** and **11b**). Finally, we also now include data using blastoids (i.e. blastocyst-like structures derived from ESCs) that demonstrate functional differences between the two *Snhg26* isoforms (**Fig. 8e-h**). Therefore, on top of using a very deep lrRNAseq dataset, we used RT-PCR, other short-read RNAseq datasets, and functional assays to validate isoform switching for *Snhg26* and the role of its isoforms in pluripotency.

*4. Figure 4A: are the numbers referring to the exact same alternative splicing events or to the genes undergoing alternative splicing during reprogramming? This should be clarified.*

The numbers refer to the same genes, but not to the exact same alternative splicing events, as this analysis would prove tricky in reason of the different types of datasets compared (i.e. short-read vs long-read RNAseq). We thank the reviewer for pinpointing the lack of clarity in this figure, and this is why we added more precise information in both the figure legend and Methods section to improve understanding for the readers.

*5. Figures 4E-F: can the predicted functional effects of FYN splicing switch be validated in the transcriptome datasets (e.g. changes in expression of downstream targets of FYN)?*

For determining downstream effects on FYN targets, we assessed the change in expression of genes positively or negatively correlated with *Fyn* expression on DepMap (12 genes for each set, see "Fyn-targets-expression" tab in **Supplementary Table 4**). We show that expression increases for negatively-correlated genes during OKS reprogramming, consistent with the observed increase in inactive *Fyn* isoforms in early reprogramming. This was added as **Fig. 4i**, and suggest that increase of an inactive isoform of *Fyn* at the beginning of reprogramming corresponds accordingly with the regulation of its target genes.

*6. Figure 5A (and others): it would be good to clarify what upregulation or downregulation of isoforms means. For example, for cassette exons, increases in exon inclusion will be accompanied by concomitant decreases in exon skipping and vice versa. How are these two events counted in these graphs? The same for Alt 5' or 3' ss or intron retention events.*

We thank the reviewer for pointing this out and apologize for the lack of clarity. Regarding **Fig. 5a** and others, when we discuss isoform expression, we are simply looking at isoforms, regardless of the type of splicing event implicated. Instead, we identify the isoform corresponding to each read, to quantify expression of full-length isoforms. As for **Fig. 5a** in particular, it represents the number of isoforms for which expression was significantly up- or down-regulated (fold change $\geq 2$ or $\leq 0.5$ and adjusted p-value $\leq 0.05$) between two timepoints of the reprogramming process.

For alternative splicing events, they are treated separately than isoforms in those figures. Evolution of exon inclusion/skipping through reprogramming phases is better reflected in **Fig. 3c**, where we quantify individual types of splicing events significantly more or less detected (change of PSI of ≥ 10% and adjusted p-value ≤ 0.05), regardless of which isoform they come from. As an example, 953 events of skipped exons are significantly more detected in the early phase of OKMS reprogramming compard to the NPCs ("more included", meaning ΔPSI ≥ 0.1 and adjusted p-value ≤ 0.05), while 1,522 events of skipped exons are significantly less detected ("less included", meaning ΔPSI ≤ -0.1 and adjusted p-value ≤ 0.05) (**Fig. 3c**). The numbers do not indicate how many events of included or excluded exons are detected concomitantly within one isoform.

*7. Figure 5E: similar to point 5 above, can the authors validate downstream effects of changes in SRSF2/SRSF3 levels by looking at changes in alternative splicing of known target genes of these factors?*

We again thank the reviewer for suggesting this validation. We performed this analysis as per their request and included it in **Supplementary Fig. 9i**. Based on our new isoform quantifications, only *Srsf2* showed significant isoform switch during reprogramming with both the ΔPSI-based method and the differentially expressed isoforms-based method, so in this revised version we only focused on *Srsf2*. As the reviewer suggested, we investigated the outcome of *SRSF2* target mRNAs from Han *et al.* (Mol. Cell 2017) upon reprogramming. We see that the first half of reprogramming is associated with an increase in *SRSF2* target isoforms that do not encode full-length proteins (**Supplementary Fig. 9i**): NMD isoforms in OKMS reprogramming, and non-coding isoforms in OKS reprogramming. Interestingly, when *Srsf2*'s CDS isoforms become more prevalent in late reprogramming (**Fig. 5i** and **Supplementary Fig. 9f**), we observe a concomitant decrease in NMD/non-coding isoform levels for the target mRNAs. This shows that changes in *Srsf2* isoform levels has downstream effects on expression of its target genes.

*8. Page 13: I am not sure I follow the argument that because "there is a negative selection against lncRNAs to undergo IS", those "lncRNAs that undergo IS may harbor important functions". The negative selection may not be as strong as not to allow some lncRNAs to undergo IS switches without necessarily implying important functional roles for the switch.*

We thank the reviewer for his comment. We agree that this statement was not clear. We now show, as per reviewer 4's request (point 7), that there is a general negative selection against non-coding isoforms (whether they are coming from lncRNAs or from protein-coding genes) and an increase in isoforms coding for proper protein domains (i.e. PFAM). We show these results in **Fig. 4j-k** and **Fig. 5f-g** and demonstrate that during early reprograming, there is significant increase in transcripts containing a annotated functional protein domain and that are undergoing isoform switch (**IS**), and this is followed by an concomitant decrease in non-coding transcripts of protein coding genes (**Fig. 5f**). This suggests that IS is involved in the functional regulation of protein-coding genes. We also observed that lncRNAs undergoing IS were significantly less prevalent than what would be expected by chance (**Fig. 5d**). As such we reasoned that if the logic fits for protein coding genes, where IS during reprogramming selects for isoforms that code for the functional domain, then we would expect that the same applies for lncRNAs and that IS of lncRNAs would

select for functional lncRNA isoforms. We modified the end of the "Isoform switching selectively regulates non-coding isoforms during early reprogramming" section to better reflect this statement.

*9. Figures 6A-B: the selection of the 19 candidates (p-13, line 31) should be better explained and justified in the text. Panel B: the 2 lncRNAs selected were the only ones expressed in iPSC cells or (p.14, line 1) the ones particularly highly expressed? How was this threshold established?*

We thank the reviewer for highlighting the need to clarify this figure. Because end of section 5 ("Isoform switching selectively regulates non-coding isoforms during early reprogramming") highlights our wish to investigate IS in lncRNAs, and not protein-coding genes, we removed from **Fig. 6a** additional information regarding other gene biotypes, and kept only the numbers relevant to lncRNA genes. Also, we changed the nomenclature of the two lists of genes merged in **Fig. 6a** so that it reflects better how they were selected: the "ΔPSI events-based method" refers to **Fig. 3d**, and the "DEIs-based method" refers to **Fig. 5b**. The manuscript was updated to keep the same nomenclature as the figure and to refer to the corresponding figures. We hope this will clarify our analysis process for both the reviewer and future readers.

Regarding the selection of the 2 lncRNA candidates, those were chosen based on the expression profile throughout two MEF reprogramming datasets (Hussein *et al.,* Nature 2014; Golipour *et al.,* Cell Stem Cell 2012) AND comparison of their expression levels in iPSCs and MEFs (see pipeline in **Fig. 6b**). Here, we chose to look at datasets from MEF reprogramming, because datasets from NPC reprogramming were not available. Also, MEFs constitute a more differentiated cell type than NPCs (which are multipotent stem cells), and the difference of expression of the lncRNA candidates between the starting cell type and the final iPSCs would be more striking. We identified *2700038G22Rik* (i.e. *Snhg26*), *2010204K13Rik*, *Gm11944*, and *Mir17hg*. These are the only four lncRNAs where expression increases throughout both reprogramming datasets and remains higher in iPSCs than in MEFs (**Fig. 6c** and **Supplementary Fig. 10a-b**), suggesting a possible role in the acquisition of the pluripotent state. The expression of other candidates either remained unchanged or even lower, in iPSCs compared to MEFs, for both reprogramming datasets that were examined. Moreover, their expression levels seem to vary and often decrease during reprogramming. This suggests that they may not be as important for pluripotent stem cells. Finally, only *2700038G22Rik* (i.e. *Snhg26*) and *Mir17hg* were annotated as human lncRNAs and showed a conserved pattern of expression in human reprogramming data (**Fig. 6e** and **Supplementary Fig. 10c**).

To summarize, the 2 lncRNAs were chosen because their expression is consistently higher in iPSCs than in MEFs and increases during reprogramming, and is conserved in human reprogramming. We modified the manuscript and **Fig. 6b** so that it reflects better this selection process. We chose to focus on *Snhg26* since nothing is known about its function, whereas *Mir17hg* may function through the miRNAs it harbors, and those miRNAs have already been shown to regulate pluripotency (Foshay *et al.,* Dev. Biol. 2009; He *et al.,* PLoS One 2014). This is now highlighted in the manuscript.

*10. Figure 7: the exact snhg26 isoform used in the overexpression experiments and its choice given the IS described in Figure 6 should be clearly explained and justified.*

The *Snhg26* isoform used for the overexpression experiments was chosen based on the two isoforms that undergo isoform switching during early reprogramming, i.e. *Snhg26.216* and

*Snhg26.217. Snhg26.217* is the one that increases during the early phase of reprogramming, while *Snhg26.216* decreases (**Fig. 6g**). Also, *Snhg26.217* was the predominant isoform in single-cell data of reprogramming from Kim *et al.* (Cell Stem Cell, 2015) (**Fig. 6h**) and cells expressing this isoform correlated with expression of ESC-like genes in transitional reprogramming cells (**Fig. 6i**). Thus, we reasoned that *Snhg26.217* may have the potential to promote the pluripotent state. This is why we decided to chose this isoform for the overexpression experiments. We thank the reviewer for raising this important point, and we modified all the figures and their legends so that this information is clearly depicted for the readers.

Additionnally, we now show that loss of *Snhg26* decreases the size, cell number, and the number of SOX2+ cells (marker of inner cell mass-like cells, i.e. pluripotent cells) in *in vitro Snhg26* knock out (KO) blastoids models (**Fig. 8e-h**). Only the *Snhg26.217* isoform is able to rescue the formation of blastoids and the inner cell mass-like cells in *Snhg26* KO blastoids. Moreover, the expression of *Snhg26.217* is markedly increased during the genome activation phase (2-cell stage) of embryo development compared to *Snhg26.216* (**Fig. 8d**). Put together, our results demonstrate that *Snhg26*, more specifically the *Snhg26.217 isoform*, is important in acquisition of pluripotency.

*11. Given the relevance to prove effective reprogramming, I would recommend to bring the embryo data now shown in Extended Data of Figure 8H to the main Figure 7.*

We thank the reviewer for this comment. Reviewer 4, point 1, expressed the need to validate our obtained iPSCs further using supplementary teratoma or chimera formation assays. Thus, validation with adult chimeras is now shown in **Fig. 1d** and **Fig. 7j**, wheras embryo chimera and teratoma are displayed in **Supplementary Figures 13g-h** and **14**. We modified accordingly the corresponding figure legends as well as their reference in the results sections.

*12. Figure 8F: it would be good to include also quantification data of colony sizes in addition to colony numbers.*

We agree with the reviewer that analysis of the colony sizes should have been added to Figure 8f. We proceeded with their request and added a graph now in **Fig. 9i**, showing that knock-down of *SNHG26* decreased the average size of iPSC colonies based on OCT4 staining. We think that this result better supports our claim that "iPSC colonies with *SNHG26* knock-down showed a lower density and size compared to reprogramming without and with control ASOs" in p.30 line 13 of the manuscript. Reference to the figure was adequately added in the corresponding section of the text, and the figure legend was updated.

*Juan Valcarcel*

We would like to thank Dr. Valcarcel for the constructive comments and suggestions. We believe Dr. Valcarcel helped increase substantially the quality of the manuscript.

**Reviewer #2 (Remarks to the Author):**

*Authors hereby present a very interesting and sound model for cellular reprogramming, and generate a valuable lrRNA-seq dataset covering the process*

*across different timepoints. Alternative isoform expression is a key regulatory mechanism for many biological processes, including differentiation, but it is often overlooked or only analyzed from an exon-centric perspective. In this context, this research is both timely and relevant.*

*Therefore, my comments do not intend to put the biological insights obtained from the study into question, but improve the decisions made during long-read data processing and transcriptome reconstruction. The usage of SQANTI3 for transcriptome curation in this study shows that authors are aware that lrRNAseq data is rich in artifacts. However, more extensive analyses and filtering should be performed before trusting the data, especially regarding novel isoforms. I trust that these suggestions will help authors improve their manuscript and contribute to enhance the relevance and soundness of their discoveries.*

We would like to thank the reviewer for their input and thoughtful suggestions and for the time dedicated to reviewing our manuscript.

*Main comments:*

*1. The transcriptome presented in the manuscript is very large, and high percentage of novel isoforms is concerning, particularly when it comes to NNC transcripts. I suggest the following measures are taken to ensure that the isoforms included are realiable and provide readers with a detailed account of the transcriptome's properties:*

We would like to thank the reviewer for pointing this out and for their suggestions. One point that was not made clear in the first version of the manuscript is that starting from Figure 2e of the original manuscript, we were using a filtered down version of this transcriptome containing ~280,000 isoforms. This was explained in Extended Data Figure 3a of the original manuscript (now **Supplementary Fig. 2**) but was not evident in the main text. We apologize for the confusion and thank the reviewer for their input. Considering ~280,000 isoforms, comparison to other published work shows that this number of isoforms is not extremely large for a time-course experiment with 10 time points, each including several distinct cell states. For example, the original SQANTI3 manuscript (Pardo-Palacios *et al.,* Nature Methods 2024), analyzing only one human cell line, found ~140,000 isoforms after filtering, while Leung *et al.* (Cell Reports 2021) showed from ~18,000 to 46,000 isoforms per sample in mouse and human brains.

*1.A. Even though polyA motif and TSS (i.e. CAGE data) data are incorporated, it is important to look at quality metrics that are specific to the transcript area presenting novelty (in this case, the novel splice junctions). This could be any feature in SQANTI3 related to junction coverage by short reads, FL counts or even expression. Further evidence needs to be supplied to validate these novel transcripts, filtering those cases where anomalies are found (i.e. novel junctions with no coverage). Validation with short reads would be ideal and, if no compatible short read dataset is available, a transcript expression threshold should be applied within the rules filter to ensure that*

*the novel isoforms are reliable. Detection of the junctions in a minimum percentage of the replicates would be another option to validate these potentially novel isoforms.*

We thank the reviewer for bringing these important points forward. Our SQANTI3 filter kept only isoforms with canonical splice sites (as the default rules require canonical splice sites to be present when no short reads are supplied). However, we agree that this is not a sufficient parameter to fully validate the potentially novel isoforms. Since we have no comparable short-read datasets, we instead investigated publicly available RNAseq data from a wide variety of sources, to assess if these junctions were detectable in some cell types. We show that virtually all FSM, ISM and NIC transcripts are supported by these short-read datasets (**Supplementary Fig. 3e**). However, as the reviewer suspected, only 31% of the NNC transcripts presented in our work have all their junctions supported by short reads. However, one advantage of long-read RNA-sequencing is higher detection of repetitive elements in the transcriptome. Interestingly, of the remaining isoforms, several of the junctions lacking short-read coverage overlapped a repetitive sequence, which we show display lower detection in short-read RNAseq, transcriptome-wide (**Supplementary Fig. 3f-g**). It is a well documented issue that short reads struggle with repetitive elements such as transposable elements or low-complexity sequences (Ozsolak *et al.,* Nat. Rev. Genet. 2011; Jin *et al.,* Bioinformatics 2015; Liao *et al.,* NAR 2021; Tanudisastro *et al.,* Nat. Rev. Genet. 2024). When accounting for the presence of repetitive elements, we show that 53% of NNC isoforms have all their junctions either supported by short reads, or their lack of support explained by the presence of repetitive sequences (**Supplementary Fig. 3e**). Furthermore, out of the remaining 60,442 NNC isoforms not supported, 87% (52,835 isoforms) are coding isoforms. Looking at our deep coverage proteomics data from mouse ESCs (data added for this version of the manuscript), peptides corresponding to 3,643 additional isoforms were detected, accounting for 7% of the missing coding isoforms. This brings our total number of isoforms for which we have strong evidence to 227,314 (i.e. 81% of the 279,998 detected isoforms) (**Supplementary Fig. 3d**). Furthermore, as the reviewer proposed, we also used an expression threshold of 1 TPM in at least 3 samples to filter out low-expression isoforms. We show in **Supplementary Fig. 5a-e** that the isoforms whose junctions could not be validated have similar lengths, exon counts and expression compared to supported isoforms. We therefore believe that, considering the lack of directly comparable short-read data, it would be premature to exclude these isoforms.

Lastly, the isoforms highlighted throughout the manuscript for *Snhg26* and *Srsf2* all have their splice junctions validated (see “Classification-file” tab in **Supplementary Table 2**).

*1.B. Related to the previous point, it would be interesting to see the distribution of some relevant metrics across categories (if available), such as expression/FL count, minimum coverage (if short reads are available), length/exon number, CAGE peak and polyA motif detection, distance to TSS/TTS, etc. to better understand whether these transcripts are generated by some kind of library-related artifact, and make informed decisions to remove them. This detailed description of the properties of transcripts and the category they belong to would fit nicely as a supplementary figure, including information before and after filtering to ensure that the artifacts have been successfully removed.*

We agree with the reviewer, and we now added the requested metrics and data as **Supplementary Figures 3b-c**, **4** and **5**. These include the data mentioned in the point above but also H3K4me3 and CAGE data for identification of TSS (see answer to comment 2.B below for details). This data is now included in **Supplementary Table 2** and files used to generate these analyses are presented in **Supplementary Tables 2** and **3**, and **Supplementary Data 1** and **2**.

*2. Regarding filtering, I have several additional concerns:*

*2.A. Authors should pay attention to the default rules filter in SQANTI3, and note that it does not consider polyA motif or CAGE peak information. Per the author's description, I understand that isoforms not including a polyA motif or exhibiting RT switching are being eliminated after the rescue is run. RT switching-based filtering is already incorporated in the default rules, but it would be best to include polyA motif detection as part of the rules filter and for all isoform categories, as the removal of these transcripts prior to the rescue will surely affect how this step is performed, impacting the final composition of the transcriptome.*

We thank the reviewer for this comment. We agree that the method proposed by the reviewer is sound. In our datasets, as mentioned in our response to the above points, we used stringent criteria to filter isoforms, including but not limited to expression thresholds, splice junction validation, and polyA motif detection. Also, SQANTI3 rescue recovers less than 0.5% of the total isoforms in our hand, and we therefore believe that changes to this step will result in very minimal changes to the generated transcriptome. We believe that as the reviewer pointed out, incorporating different metrics and data sources is most likely the best option to validate isoforms and to ensure that we do not include artifacts. Also, as we demonstrate in **Supplementary Fig. 4b**, we no longer detect transcripts without a polyA motif post-filtering.

*2.B. In addition, it seems that no filtering decision is made based on CAGE peak information, in spite of using this information when running SQANTI3 QC. It would be adviseable to add this to the rules filter (via the within CAGE peak metric or using threshold of distance to the nearest CAGE peak) for all categories. Also, the details of the CAGE peak dataset being used for this purpose is not included in the relevant methods section.*

We once again agree with the reviewer that this method would work to obtain transcripts with high-confidence 5' ends. The reviewer comment inspired us to adapt a filtering strategy where we incorporate multiple data types and not only CAGE peak data offered by the SQANTI package. We followed a slightly different approach than proposed by the reviewer, and we now show that most of the transcripts included in our transcriptome have proper TSS support by CAGE-seq or other comparable datasets (**Supplementary Fig. 3b-c**). We searched for 5' support from the refTSS4 database (Abugessaisa *et al.,* JMB 2019), which includes the FANTOM5 annotations. We also included the TE-TSS database (Gu *et al.,* NAR 2024), 5' RNA-sequencing from O'Malley *et al.* (Nature 2013), which was performed during reprogramming, and CAGE-seq data from iPSCs (Fort *et al.,* Cell Cycle 2015). Reasoning that transcription start sites situated within repetitive regions of the genome could not be efficiently detected from the short-read sequencing datasets that we used, we also accounted for the overlap of repetitive elements over the TSS of our isoforms.

We show that 80% of isoforms are supported by these datasets (**Supplementary Fig. 3b**). Aiming to assess whether the remaining non-supported isoforms were real or artifacts from the library preparation, degradation or others, we evaluated both the H3K4me3 status at their promoter, as well as their TSS ratio (see illustration below).

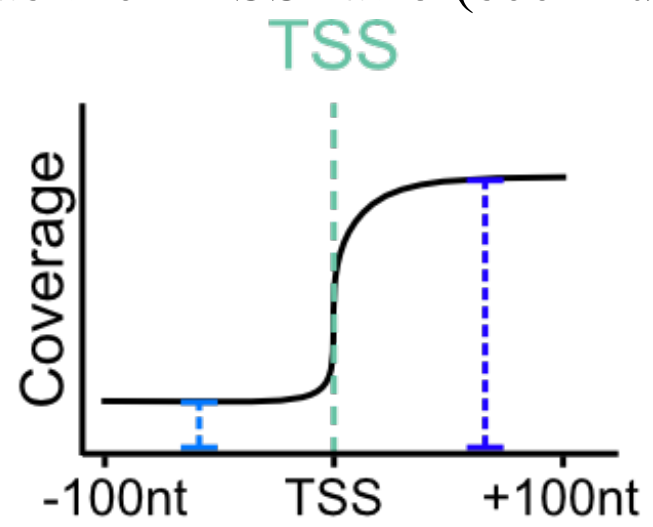


$$\text{TSS ratio} = \frac{\text{Coverage 100nt after TSS}}{\text{Coverage 100nt before TSS}}$$

A TSS ratio of 1 or below is indicative of a falsely identified isoform. Based on the TSS-ratio threshold of 1.5 established for proper isoforms by Pardo-Palacios *et al.* (Nature Methods 2024), we show that more than 2/3 of unsupported isoforms have a TSS ratio superior to 1.5 and/or H3K4me3 peaks at their TSS. Taken together, 93% of the isoforms presented in this paper show strong evidence of a proper 5' end (**Supplementary Fig. 3b**).

Similarly to the transcripts lacking junction support above, we find that transcript lengths, exon counts and expression of isoforms without a supported TSS are very similar to those of supported isoforms (**Supplementary Fig. 5f-j**). If ISM transcripts were generated by library degradation for instance, we would expect a global shortening of transcript lengths and number of exons, but seeing as this is not the case (**Supplementary Fig. 5f-j**), we opted to keep the small percentage (7%) of isoforms whose TSS is not validated. Of note, as mentioned earlier, isoforms highlighted in the manuscript are fully validated for their TSS and splice junctions. Finally, as requested by the reviewer, we have added the details of datasets used for validation of 5' ends in the Methods ("Preparation of a catalog of validated isoform 5' ends" subsection) and in the "Datasets" tab of **Supplementary Table 3**.

> *2.C. I suggest completing polyA and CAGE peak filtering with other annotation and data-based TSS and TTS quality metrics, such as the distance to the annotated TSS/TTS and the TSS ratio, as it has been shown to be relevant to remove artifacts, especially within the ISM category.*

We agree with the reviewer that these are good methods, and as mentioned above, we now added these figures as **Supplementary Figures 3b-e** and **4** (data also available in **Supplementary Table 2**).

> *Authors are referred to the SQANTI3 manuscript for ideas as to how these filtering criteria can be incorporated into a custom-made rules filter script.*

> *3. Regarding the results oultined in section 3 (i.e. Exclusive regulation of non-coding isoforms):*

> *3.A. I am curious as to why authors do not start by performing an analysis of coding vs non-coding isoforms as a whole. It might be that the results described here can be attributed to the properties of non-coding isoforms as a whole, not solely to novel non-coding isoforms. In other words, do coding vs non-coding isoform results replicate those observed for non-coding NNC?*

The reviewer raises a very interesting point. We analyzed, as the reviewer suggested, coding and non-coding isoforms in the overall dataset, and found a significant increase in coding isoforms when we compare the starting cell type (i.e. NPCs) and the middle phase of reprogramming, leading to a significant decrease in the proportion of non-coding isoforms (**Fig. 2e**). This is also seen when comparing the NPCs to the final iPSCs. However, when looking in more detail at the fraction of annotated and new transcripts, we noticed that only new isoforms (from both known and novel genes) were affected (**Fig. 2f-g**). Thus, annotated non-coding isoforms do not seem to undergo the same level of regulation as the new ones.

*3.B. It is not clear from the methods whether authors derived these results by performing multiple pairwise analyses between D0 and the different phases with edgeR. If so, the correct approach to this analysis would be to fit one GLM model per isoform using edgeR with the different phases as a covariate, deriving significant changes in isoform expression across reprogramming (using the adjusted p-value), and then test each of the coefficients for significant changes in expression. I apologize if this was the approach used in the first place, and would therefore recommend authors to include a more detailed account of this step in the relevant methods section and make the code available to ensure reproducibility and avoid confusion. In addition, I believe puting the differential gene expression and differential isoform expression analysis sections in the methods next to each other, and after the transcriptome assembly step, would help follow the text.*

We thank the reviewer for this comment, as the methods of the paper indeed lacked clarity for this section. We indeed used edgeR with a GLM model to assess significant changes in isoforms across phases. Changes in expression were considered significant with adjusted p-values $\leq 0.05$ and fold-changes $\geq 2$ or $\leq 0.5$. All relevant details have been added to the Methods section ("LrRNAseq data analysis of gene expression" and "Analysis of isoform level expression and isoform switching" subsections). Regarding the description of differential gene and isoform expression in the Methods section, we modified the order in which they appear as per the reviewer's request.

*4. I am aware that the implementation of these comments may alter some of the transcriptome-wide conclusions in the subsequent sections of the manuscript. I wish to thank the authors in advance for their time and dedication, and look forward to reading the revised version of their work.*

We are very thankful to the reviewer for this kind and noteworthy comment, and we think that following their thorough advice regarding the analysis of our dataset substantially improved its quality. We hope that the reviewer will share our opinion.

*Minor coments:*

*- Page 6, line 22-23: "Novel In Catalog (NIC)" instead of "Not In Catalog"*

We thank the reviewer for pointing out this error, and it is now corrected in the revised manuscript.

**Reviewer #3 (Remarks to the Author):**

> *This study aims to identify novel functional isoforms regulating cell fate using long-read RNA sequencing (lrRNAseq). The authors employ two reprogramming systems, OKMS and OKS, to distinguish c-Myc-dependent and -independent effects on alternative splicing (AS) and isoform regulation. They identify multiple previously undetected isoforms with dynamic expression patterns during reprogramming. Notably, non-coding isoforms are negatively selected during mid-phase reprogramming, while long non-coding RNAs (lncRNAs) undergoing IS appear functionally important for pluripotency acquisition. Finally, the study demonstrates that overexpression of the lncRNA Snhg26 enhances reprogramming efficiency. While the study comprehensively profiles long-read RNA seq during reprogramming, the manuscript is largely descriptive and functional validations requires a careful re-examination the use of controls and targeting strategies.*

We would like to thank the reviewers for their suggestions and thorough review of the manuscript.

> <u>*Major Comments*</u>
> *1. The study includes 22x R9 and 13x R10 samples. The differences in read accuracy or coverage can significantly lead to biases isoform detection. Please explain the extent of batch effects observed across the samples and how the batch effects across these sequencing versions were accounted for.*

We would like to thank the reviewers for their time and efforts in reviewing our manuscript and for raising this important concern. Because Oxford Nanopore Technologies flow cells keep evolving, we tried to keep up with this evolution during the development of the research project proposed in this manuscript. It was difficult not to get samples with differences in read accuracy and coverage. In our hands, we observed a difference in read coverage for the samples sequenced with R9 and R10 flow cells (see "Statistics-lrRNAseq-reads" tab in **Supplementary Table 1**, R9 samples on average: 2.5 million raw reads, R10: 5 million) but did not observe any detection bias in isoforms detected from annotated genes, which constitute the majority of our data (**Supplementary Fig. 6a**). We also have replicates from R9 and R10 technologies for all of our reprogramming phases, thus equilibrating any bias that may arise. Furthermore, differences in coverage are taken into consideration, as we perform normalization to the libraries and transcript sizes (TPM expression values). The difference in read accuracy between the R9 and R10 flow cell technologies has proven to be 2-3% in the literature (R9 median ~96-97% vs R10 median ~98.8-99.2% according to Sanderson *et al.,* Microb. Genom. 2023 and Ni *et al.,* Comput. Struct. Biotechnol. J. 2023). Finally, for the purpose of this rebuttal, we added 4 new R10 replicates of reprogramming samples (2 at D18 and 2 at D18 in 2i-LIF, i.e. iPSC stage) and 6 replicates of R10 ESC samples to make our dataset even more robust. Using different versions of ONT flow cell technologies and many replicates per phase thus allowed us to account for the batch effect mentioned by the reviewers.

*2. D14 and D20 OKS samples are grouped as "late phase," yet Extended Data Figure 2C shows substantial differential gene expression between them. Please clarify this point, including ONT R10 data for whole-transcriptome DE and clustering analysis (OKMS vs. OKS) could refine phase definitions and further strengthen conclusions.*

The reviewers have a valid point that OKS reprogramming D14 and D20 samples behave quite differently. We had intentionally grouped D14 with D20 in order to get at least two timepoints within the OKS late phase. Nonetheless, we agree with the reviewers that this was not ideal and thank them for pointing it out. In order to better refine phase definitions, we decided to sequence D18 from both OKMS and OKS reprogramming systems, as they would fit more adequately into the OKS late phase than D14. We performed a new analysis of differential gene expression and hierarchical clustering (see **Fig. 1b-c** and **Supplementary Fig. 1c**) based on the lists of reprogramming and pluripotency-associated genes from Hussein *et al.* (Nature 2014) (same as used in original **Fig. 1b**), taking into account our complete dataset of now 45 samples. This enabled a much more precise clustering of the samples and led us to change our sample grouping: D2 samples were conserved as the early phase of both systems ; D4 and D6 were maintained as the middle phase of OKMS ; D4, D6 and D10 were also kept as the middle phase of OKS ; however, D18 was added to D10 and D14 for the late phase of OKMS ; and D18 and D20 were defined as the late phase of OKS. We chose to exclude the D14 OKS from the grouping, as it was still clustering separately from the D10 and D18 timepoints, suggesting that it stands alone during the OKS reprogramming process. This way, D18 and D20 of OKS cluster very closely to each other and to the iPSC cluster, thus better representing the late phase of OKS reprogramming.

*3. Somatic reprogramming process is highly heterogeneous – even with Dox system (https://www.cell.com/fulltext/S0092-86741201021-5). What is the level of heterogeneity in the dataset – such as running single cell RNA-seq- during the progression of OKMS and OKS reprogramming will reveal this and further clarify whether the isoforms are stage specific vs cell type/state specific.*

We agree with the reviewer that our bulk sequencing approach does not allow us to decipher between whether isoforms are stage-specific (i.e. expressed in all cells of a reprogramming time-point) or rather enriched in a subset of cells during reprogramming (e.g. the sub-population of cells that will actually reprogram). To investigate this, we analyzed published short-read single-cell RNAseq data from reprogramming cells (Kim *et al.,* Cell Stem Cell 2015). With the caveats that short-reads are not as efficient at identifying isoform expression, and that single-cell RNA sequencing datasets generally exhibit low sequencing depths, we assessed the expression of the isoforms from our long read-built transcriptome in single cells undergoing reprogramming (**Fig. 6h-j** and **Supplementary Fig. 11c-f**). We show that not all cells that go through reprogramming express the same isoforms. For example, only a subset (< 25%) of reprogramming cells express coding isoforms from the pluripotency genes *Dppa2*, *Utf1* and *Esrrb* (**Supplementary Fig. 11e**). These three genes were shown to be predictors of reprogramming outcome (Buganim *et al.,* Cell 2012). Consequently, filtering for cells that express coding *Dppa2*, *Utf1* and *Esrrb* isoforms reveals significantly higher expression of early ESC-like or ESC-like gene sets (**Fig. 6j**). This suggests that these cells may indeed be further along the process of reprogramming.

With this in mind, we also show that reprogramming cells tend to express either *Snhg26* isoform 216 or 217 (**Fig. 6h**). Cells that express both concomitantly are rare (only 2 out of 26

reprogramming cells). Interestingly, cells that express *Snhg26.217* have significantly increased expression of ESC-like genes (**Fig. 6i**). Isoform 216 is generally associated with a trend of higher expression of earlier reprogramming genes (immediate responder genes or intermediate reprogramming genes), though this effect was not significant (**Supplementary Fig. 11d**). This adds to the results highlighted from our datasets, where overexpression of isoform 217 resulted in enhanced reprogramming.

Taken together, these results suggest cell-state specific isoforms that mark distinct sub-populations of cells during the reprogramming timeline.This constitutes an interesting point that was added to the manuscript.

*4. (Figure 2A) How does the percentage distribution of isoform categories (e.g., NNC, annotated) vary across reprogramming stages? Are certain categories underrepresented due to read coverage biases? Providing read counts per category would address detection sensitivity.*

We thank the reviewers for asking these relevant questions. To answer them, we added the sums of the transcript counts detected for each structural isoform category to the “Detailed-isoform-classification” tab in **Supplementary Table 2**. The percentage distribution of read counts per isoform categories across reprogramming stages were also added to **Supplementary Fig. 6b**, showing no variation for isoforms coming from annotated genes (i.e. FSM, ISM, NIC, NNC). As for novel isoforms from new genes (i.e. genic, intergenic, fusion, antisense), we observe a decrease at the early stages of reprogramming but these changes may be due to low number of isoforms found (4% of all isoforms, **Fig. 2a**). Therefore, we believe that there is no bias towards certain categories because the read coverages are similar.

*5. (Section 3, Line 27) How many "isoforms from novel genes" were analyzed? Are they exclusively non-coding, or do some retain coding potential?*

Isoforms from novel genes correspond to 11,169 transcripts, which is indicated in **Fig. 2a**. These are both coding (4,648 isoforms) and non-coding (6,521 isoforms), so many still retain coding potential. This is described in now section 2 (“Unprecedented isoform diversity and tight regulation of non-coding isoforms during reprogramming”). The **Fig. 2e-g** now presents the data by segregating coding from non-coding isoforms for the isoform categories. We arranged the text of the manuscript to make this clearer. We hope this will answer the reviewer’s questions.

*6. Defining novel transcripts requires strong transcript evidence. Systematically annotate novel transcripts with well-annotated 5’ support such as FANTOM and use orthologous tools such as BAMBU (https://www.nature.com/articles/s41592-023-01908-w) to provide confidence to these novel transcripts as opposed to potential spurious or degraded transcripts. Moreover, majority of lncRNAs are non-poly-adenylated, yet many were discovered using poly-dT reverse transcription library prep in ONT. Please explain to what extend the newly discovered lncRNAs are polyA+ and polyA- and verify them.*

We agree with the reviewers that validation of novel transcripts is a critical step. To increase confidence in our novel transcripts we followed the reviewers’ advice and used 3 orthologous

tools: Bambu, IsoQuant and StringTie, three well-supported isoform detection tools currently available (Pardo-Palacios *et al.,* Nature Methods 2024) (**Supplementary Fig. 3h**). We found that a large proportion of isoforms detected by our method, or by either Bambu, Isoquant or Stringtie, are not found by the other algorithms when compared to each other, averaging to less than 50% overlap between each other (**Supplementary Fig. 3h**). This highlights that many currently available long-read transcriptome generation tools have their limitations. This was further emphasized in Pardo-Palacios *et al.* (Nature Methods 2024), demonstrating that each tool has its advantages and disadvantages. As described for reviewer 2 (points 1A-B and 2B-C), we used multiple isoform quality metrics (**Supplementary Figures 3**, **4** and **5**) to evaluate and increase confidence in our identified isoforms. These include for TSS: TE-TSS and refTSS4 database, which includes the FANTOM5 annotations, 5' RNA-sequencing (O'Malley *et al.,* Nature 2013) and CAGE-seq data on reprogramming and iPSCs, H3K4me3 status at relevant promoters, TSS ratio, and for splice junctions: Short-read RNAseq, presence of repeated elements, and mouse ESC proteomics data for coding isoforms. Additionally, **Supplementary Fig. 5** shows similar features between supported and unsupported isoforms, indicating that filtering the isoforms that could not be validated is premature at this stage.

Concerning the second part of the comment on polyadenylation of lncRNAs, this point may have not been clear in the manuscript. Studies now show that the majority of lncRNAs are polyadenylated, and this is probably due to the continuous discovery and annotation of new lncRNAs and non-coding transcripts. For example, Yu *et al.* (NAR 2026) show in PolyA_DB v4 that 75% of the 16,909 lncRNAs included in the hg19 Ensembl release 102 of the human transcriptome were poly(A)+. For mouse mm10 (Ensembl release 102), 69% of the 9,981 lncRNAs are poly(A)+ (Yu *et al.,* NAR 2026). Therefore, it is not surprising that the ONT library preparation allows for sequencing of these polyadenylated transcripts. Also, one of the novelties of our study is that expression of these polyadenylated non-coding transcripts could in part counteract the expression of functional isoforms, thus uncovering new mechanisms of gene regulation that are yet to be discovered.

*7. Are there shared non-coding isoforms between OKMS and OKS (Figure 2F)? Identifying these could reveal c-Myc-independent regulatory mechanisms.*

We thank the reviewers for their comment. We now added information of genes shared between OKMS and OKS in the manuscript for IS genes only (**Supplementary Fig. 9d**). We did so because our data suggested that IS is involved in the functional regulation of protein-coding genes (**Fig. 5**), and not specifically just non-coding.

However, as per the reviewer's request, we added the graph below to show that there are multiple differentially expressed non-coding isoforms (DEIs) that are shared between OKMS and OKS during the course of reprogramming:

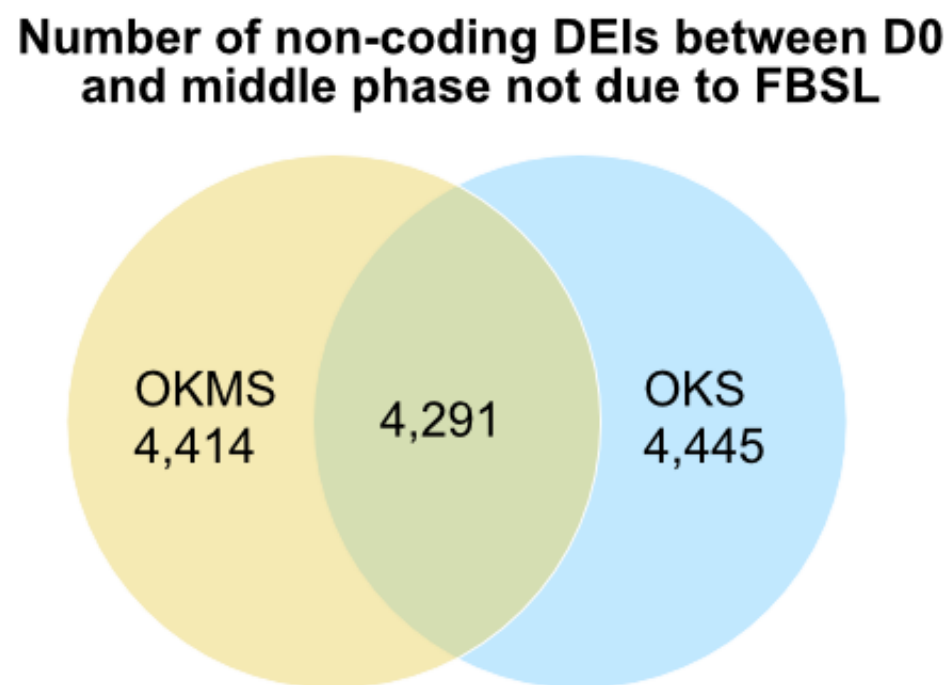


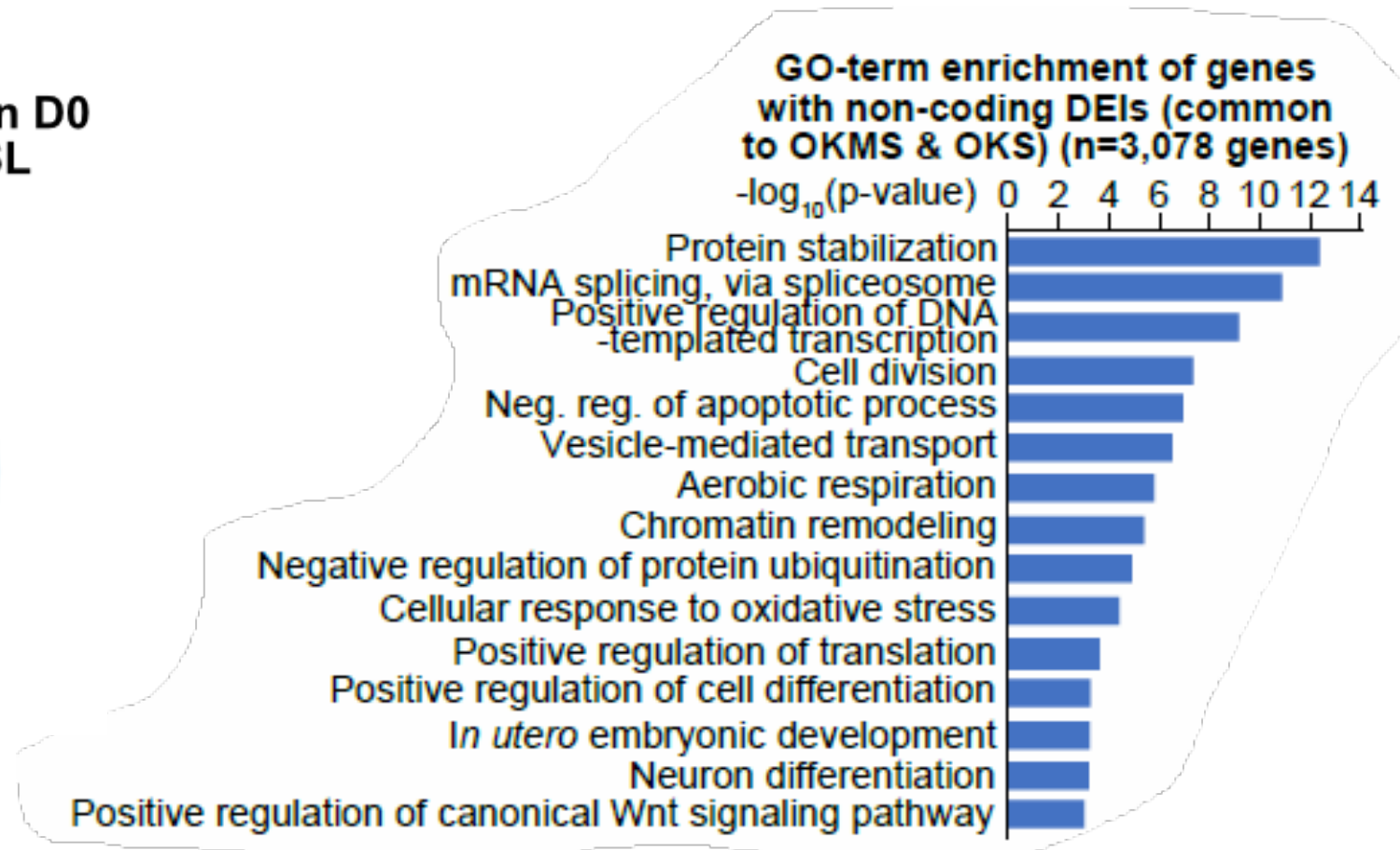


In fact, the GO-terms on the genes related to those isoforms are the same as the ones that we observe for genes undergoing IS, which are genes involved in protein stabilization and translation, RNA splicing regulation, apoptosis, regulation of transcription, mitochondrial electron transport, regulation of cell cycle, which are all important processes for the completion of the reprogramming process.

*8. The authors use both ΔPSI and TPM-based DEIs to define Isoform Switching. Are the results consistent between methods? A direct comparison would validate robustness. What are the functional implications to make the manuscript less descriptive.*

We apologize to the reviewers for not making this clear enough in the first submission manuscript, but we did compare the two methods: In **Fig. 5e** from this new submission, we showed that 12,086 genes undergo isoform switch based on the dPSI events-based method. With the TPM-based DEI method, which is generally more stringent, we identified 2,374 genes undergoing isoform switch, and the overlap between the two methods was of 74% (i.e. 1,766 genes). The 26% of genes not overlapping can be explained by the fact that the dPSI-based method, which is commonly used, is relative and indicates which isoform becomes more abundant compared to another one, by a 10% (dPSI ≥ 0.1 or dPSI ≤ -0.1) threshold. Therefore, it results in several thousands of events. On the other hand, the isoform expression-based method relies on a fold change of 2, and actually incorporates directionality of the expression change (i.e. up-, down-, "up-down", or "down-up" regulation) to determine if there is isoform switching. Thus, the number of events is more limited and more stringent. A combination of isoforms identified by both methods thus gives a high-confidence list of 1,766 genes subjected to isoform switching. This list was used to identify candidate genes undergoing isoform switching (e.g. *Srsf2* and *Snhg26*).

To make the manuscript less descriptive, we incorporated more data on the candidate lncRNA *Snhg26*, demonstrating that isoform switching analysis identifies functionally distinct isoforms. We find that the isoform *Snhg26.217,* compared to *Snhg26.216*, is prevalent in early embryo (**Fig. 8d**) and is important for acquisition of pluripotency, as demonstrated by our reprogramming (**Fig. 7** and **Supplementary Figures 12** and **13**) and blastoids (**Fig. 8e-h**) experiments. We would also like to highlight to the reviewers that this is paper is a research resource of the first complete dataset of long-read RNAseq on different cell types and somatic reprogramming. It can be used by other researchers to study in detail isoform diversity from both coding genes and lncRNAs, in different

types of stem cells and during cell fate change, and to assess functional outcomes. Moreover, in the new version, we minimized the very descriptive parts by adding more global analyses. For example, in answering points 5 and 7 raised by reviewer 1 and minor points 7 and 8 raised by reviewer 4, we provided a more global view of the functional impact of isoform switching on genes during reprogramming (**Figs. 4j-k, 3e**, **5f-g** and **Supplementary Figures 8** and **9i**). We hope that the reviewers will find this part more concrete (see sections 4-6 of the manuscript for more details).

> 9. What is the link between AS events and NNC isoforms? Do NNC isoforms originate from genes with AS events? Overlap analysis (e.g., Venn diagrams) could clarify whether AS directly generates NNC transcripts.

A direct link between the AS events and the NNC isoforms does indeed exist, as NNC isoforms correspond to new isoforms from known genes but coming from novel splice junctions, i.e. not annotated before, and multiple AS events could give rise to one NNC isoform. Therefore, it would be difficult to create a Venn diagram that would properly demonstrate this point.

> *10. (Section 5) How does exon skipping in MBNL2 correlate with its isoform expression? Is the loss of protein-coding function a direct IS effect or a secondary stress response? Controls (e.g., stress-induced splicing in non-reprogramming cells) would help establish causality.*

We answered the reviewers' question by adding *Mbnl2'*s isoforms expression to **Supplementary Fig. 8a**. From global gene expression data (**Figures 3a** and **4d**) during OKMS and OKS reprogramming, *Mbnl2* expression increases between NPCs and D2, to then remain stable until late/iPSC phase where it decreases. From *Mbnl2* isoforms expression (**Supplementary Fig. 8a**), we show that this increase is mostly due to an elevated level of the *Mbnl2* isoform without exons 6 and 9. This is consistent with our splicing event analysis (**Fig. 4c**), where we show that exons 6 and 9 get less included for both OKMS and OKS between the NPC and early stages, leading to a less functional MBNL2 protein (**Fig. 4b**). Thus, the increase in the isoform with alternative splicing events leading to exclusion of exons 6 and 9 leads to a less active form of MBNL2 during early reprogramming. However, inclusion of these exons increases back between early and middle phases of reprogramming. We show that expression of the isoform excluding both exons 6 and 9 (i.e. the non-functional isoform) inversely decreases between the early and middle phases of reprogramming, thus correlating with the exon 6 and 9 inclusion data from **Fig. 4c.**

Regarding the loss of protein-coding function, we cannot confirm whether it is due do a direct effect of IS or something else. We believe that it would be outside the scope of this manuscript to assess if IS could be due to a secondary stress response. We believe that it is a way for cells to regulate gene function. Interestingly enough, a study published on *Mbnl2* isoforms during human myogenic cells differentiation showed a decrease of the isoform containing the exons 5 and 8 (corresponding to exons 6 and 9 in mice) (André *et al.*, PlosOne 2019). In reprogramming, which could be considered as the opposite process to differentiation, we show an increase in the isoform with exons 6 and 9 during the generation of the iPSCs, which may indicate that the process of gene regulation through isoform switching is not limited to reprogramming. Thus, we believe that this change in isoform is more a way for cells to regulate cell state.

*11. In functional validation of Snhg26, is Snhg26 or its specific isoform (G38722.217) overexpressed? To test causality, perturbing splicing factors could directly determine whether IS drives reprogramming outcomes.*

We thank the reviewers for asking this question, as our lack of clarity on this matter was also raised in point 10 of reviewer 1. We overexpressed the *Snhg26.217 (G38722.217)* isoform of *Snhg26* during the functional validations in link with reprogramming. However, the experimental validations requiring knocking-down *Snhg26* relied on methods that knock-down the global *Snhg26* gene expression, and are not isoform-specific. As explained in more details in the reviewers' point 13 below, we now added data where both *Snhg26.217* and *Snhg26.216* isoforms are overexpressed and compared in a early embryo development blastoid model. The nature of the overexpressed isoforms is now better explained throughout the manuscript.

Although assessing the effect of perturbing splicing factors on IS would be very interesting to study, we believe that this is outside the scope of the current manuscript. Indeed, altering splicing factors in a complex system such as reprogramming will without doubt affect the outcomes of the process, as this has already been extensively studied in the literature (Ohta *et al.,* Cell Rep. 2013; Cieply *et al.,* Cell Rep. 2016; Vivori *et al.,* Genome Biol. 2021).

12. c-Myc-Independent Isoform Switching. Were common IS events between OKMS and OKS systems analyzed? This could reveal splicing regulation independent of c-Myc's proliferative effects.

We thank the reviewers for suggesting this analysis. We found that 522 genes were common to the list of genes undergoing isoform switch in the OKMS (n=2,081 genes) and OKS (n=815 genes) systems, representing an overlap of 64%. Among the common genes, many were involved in RNA splicing, apoptosis, cell cycle, DNA repair and protein translation and modifications (**Supplementary Fig. 9d**). This has been added to the manuscript.

*13. Targeting Snhg26 with CRISPRi prevents transcription at the promoter – thus not able to distinguish the role of transcript isoforms versus the gene. Since the author's work stems from the fact that there are isoform specific effects during reprogramming, the authors should functionally validate these claims using a method that can target the isoform more specifically. This is further noted using the ASO where the gene is suppressed as opposed to transcript specific effects. Please consider an experiment to selectively target one isoform and not the other to support the claim that isoform is causal to reprogramming rather than the gene itself. Further note that ASOs can interfere with nascent/primary transcript, thus they are not selective in suppressing specific isoforms. Splicing switching oligos or CRISPR designed to remove specific exons can clearly demonstrate the functional specificity of Snhg26 isoforms.*

We agree with the reviewers that the CRISPRi approach used in our manuscript does not make the distinction between the transcripts. However, it was very difficult to target specifically the 217 and 216 isoforms, even with splicing switching oligos. This is because they share their complete sequence except for an 800 nucleotide-long sequence within exon 4 (see schematic below). From this sequence, only 245 nucleotides (sequence highlighted in grey) are not derived from repeated elements: Indeed, the 3' and 5' sections adjacent to this 245 nts sequence are derived from RLTR48B and SINEB1 transposable elements, which are highly repeated across the mouse genome (DeBarry *et al.,* Mol. Biol. and Evol. 2006; Kass *et al.,* Genomics 2008). On the further 5' adjacent sequence, it corresponds to a region that is duplicated in the mouse genome, within the *AI506816* gene on chromosome 5, and the *ENSMUSG0000012084* gene on chromosome 15 (see schematic below). Thus, designing oligos to knock-down the 216 transcript would require using oligos that span a repeated sequence, whether it comes from gene duplication or a transposable element. Oligos specific to the 217 transcript could be designed only in the small region of 245 nts sequence that is unique, which we found to be difficult to design oligos specific to this sequence. Thus, designing oligos completely specific to one or the other isoform proved to be impossible.

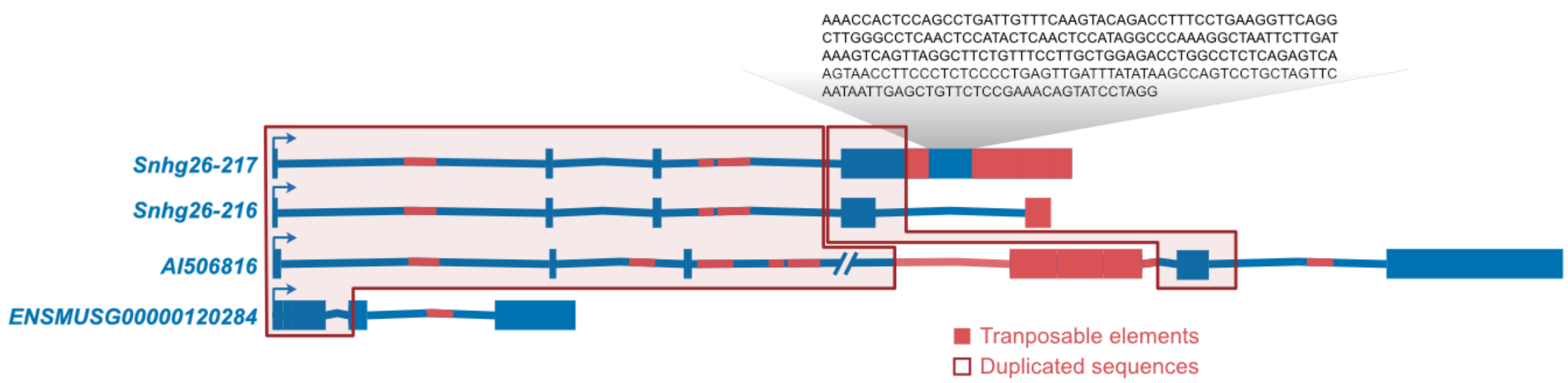


Despite these concerns, to validate the function of each isoform of *Snhg26*, we generated a CRISPR-Cas9 ESC line, where we knocked-out the 4$^{th}$ exon (last) of *Snhg26*, which represents 92.5% of its whole sequence*,* leaving only the promoter sequence of the gene intact to avoid interfering with potential regulatory domains within this region of the genome. From these cells, we made stable lines with doxycycline-inducible expression of either the 217 or 216 transcripts. We next used those *Snhg26*-KO lines to rescue the 217 or 216 isoforms expression during the generation of blastoids (i.e. in vitro models that mimic early blastocysts). KO of *Snhg26* resulted in smaller blastoids lacking the inner cell mass (ICM)-like structure, which is important for derivation of ESCs. We also found that only the *Snhg26.217* isoform rescued the size and growth of blastoids and the formation of an ICM-like structures in blastoids (**Fig. 8e-h**), suggesting that *Snhg26.217* is important for acquisition of pluripotency. Finally, we also assessed the expression of both *Snhg26* isoforms in single-cell RNAseq datasets of reprogramming from tail-tip fibroblasts (Kim *et al.*, Nature 2008), and found that the *Snhg26.217* isoform strongly correlates with expression of ESC-like genes compared to the *Snhg26.216* isoform in single cells undergoing reprogramming (**Fig. 6i** and **Supplementary Fig. 11d**).

<u>*Minor Comments*</u>

*1. CPAT Analysis Thresholds. The use of –min-orf 10 (default: 75) may inflate false-positive coding predictions. Justify this threshold or provide validation (e.g., ribosome profiling) to confirm coding potential.*

Again, we thank the reviewers for raising this concern. We chose this threshold because we aim at identifying transcripts that can lead to small peptides, not only full proteins, as it has been shown that peptides can also be functional (Slavoff *et al.,* Nat. Chem. Biol. 2013; Chen *et al.,* Science 2020; Zhu *et al.,* Mol. Cell 2026). Optimal detection of peptides in mass spectrometry data is often in lengths of 8-15 amino acids (1 to 3 kDa) (Fricker, J. Am. Soc. Mass Spectrom. 2015). This is why we felt it was better to use a threshold of 10 amino acids, to make sure that the proportion of transcripts we classify as non-coding cannot lead to translation into a peptide. We hope that this clarifies our choice to the reviewers.

*I co-reviewed this manuscript with one of the reviewers who provided the listed reports. This is part of the Nature Communications initiative to facilitate training in peer review and to provide appropriate recognition for Early Career Researchers who co-review manuscripts.*

We would like to thank both reviewers for their time and effort in reviewing our manuscript, and we highly appreciate their input, which has improved our manuscript.

**Reviewer #4 (Remarks to the Author):**

*The reviewer has carefully evaluated the manuscript entitled "Long-read RNA sequencing identifies non-coding isoform switching as a regulator of cell fate" by Fort et al. This study employs long-read RNA sequencing (lrRNAseq) to analyze RNA isoform diversity during the reprogramming of mouse neural progenitor cells (NPCs) into induced pluripotent stem cells (iPSCs). A large number of novel splicing isoforms and isoform switching (IS) events were identified, and a novel long non-coding RNA (lncRNA), Snhg26, was found to promote reprogramming via isoform switching and maintain pluripotency in both mouse and human embryonic stem cells, thus revealing the key role of isoform diversity in cell fate regulation. Overall, this study pioneers the application of long-read RNA sequencing to dissect alternative splicing and isoform diversity during reprogramming, an innovative approach that provides a new perspective for understanding the reprogramming process. However, the reviewer requests that the authors address the following revisions before a final decision on publication is made.*

We would like to thank the reviewer for the time dedicated to reviewing the manuscript and for their suggestions and thorough review of the manuscript.

*MAJOR POINTS*

*1. The reprograming efficiency and the quality of the iPSC.*

*The reprogramming system employed in this manuscript exhibits low efficiency, which may compromise the assessment of the actual efficacy of the reprogramming process and, consequently, the validity of the main findings. It remains unclear whether the reported phenotypes would persist if a more efficient reprogramming system were adopted. Furthermore, rigorous functional validation of the resulting induced*

*pluripotent stem cells—such as teratoma and chimera formation assays—is necessary to confirm the reliability of the reprogramming experiments.*

We thank the reviewer for their comments and for highlighting the point on the quality of iPSCs. We have now included full characterization of the iPSCs from our experiment, which chimera formation assays and teratomas, as per the reviewer's suggestion. Our iPSC lines demonstrate high level of chimerism (10%-60%, **Figures 1d** and **7j** and **Supplementary Fig. 13g-h**) and differentiate to all germ layers in teratoma assays (**Supplementary Fig. 14**).

Regarding the efficiency of reprogramming, we made the choice not to alter the reprogramming kinetics or trajectory by using a component that would significantly modulate its efficiency (e.g. overexpression of extra other genes aside from OKMS; or knock-down of genes that enhance reprogramming through increased proliferation). This is also why we used both OKMS and OKS reprogramming factor combinations, so that we can focus on features that are common to the two systems and thus required for reprogramming. Moreover, we believe it could be difficult to see an increase in the reprogramming efficiency when overexpressing *Snhg26* if the reprogramming efficiency was too efficient to begin with. Also, we found that *Snhg26* lncRNA enhances reprogramming efficiency with different systems and reprogramming factor combinations (see **Fig. 7g** and **Supplementary 13a-f**). Finally, even though we agree with the reviewer that different reprogramming efficiencies could alter to some extent the finding, the reproducibility of the *Snhg26* overexpression result suggests that the main findings of our paper would be extensively similar to the ones we present, if we were to use a more efficient systems. Also, to our knowledge, our OKS reprogramming system is very efficient for only using 3 reprograming factors.

Compared to other more efficient systems such as ones using multiple reprogramming factors and MEFs or B-cells, our system is quite robust as we were able to validate 90% of the genes undergoing AS events detected in the Cieply *et al.* (Cell Rep. 2016) and Vivori *et al.* (Genome Biol. 2021) data (**Fig. 4a**), demonstrating that our NPC reprogramming system is very similar to MEF reprogramming in terms of alternative splicing regulations. We also showed that 72% of the early, immediate responder, intermediate, and ESC-like genes from Hussein *et al.* (Nature 2014) were detected in our dataset (**Supplementary Note 1e**), and 22% of the lncRNAs we identified in **Fig. 6A** (i.e. 10 lncRNAs out of our 45 candidates) are also found in Hussein *et al.* (Nature 2014), Golipour *et al.* (Cell Stem Cell 2012) or Guttman *et al.* (Nature 2011) datasets. This suggests that our reprogramming system is very similar to MEF reprogramming in terms of gene expression. Moreover, the reprogramming systems from these studies have different kinetics, starting cell types (MEFs and B cells) and efficiency than our system. Therefore, it shows that gene and isoform expression in our system behave very similarly to systems that may have different efficiencies.

*2. The phenotypic and molecular mechanisms of snhg26 in somatic cell reprogramming.*
*The manuscript does not clearly define the key phenotype attributed to SNHG26 during reprogramming. The authors claim "Snhg26 lncRNA enhances the reprogramming route to pluripotency", yet it remains ambiguous whether this reflects an increase in clonal reprogramming efficiency or an acceleration of reprogramming kinetics. More detailed analyses and rigorous demonstrations are required to resolve this distinction.*

We thank the reviewer for this remark. Based on our results, the effect of *Snhg26* overexpression during reprogramming is due to an acceleration of reprogramming kinetics. To address this, we added several panels to highlight this effect, and we thank the reviewer for suggesting it.

First, we show that the *Snhg26*-induced increase in reprogramming is reproducible across several reprogramming systems (**Supplementary Fig. 13a-f**), whether they rely on transient transfections (**Supplementary Fig. 13c-e**) or stable cell lines (**Supplementary Fig. 13a-b**,**f**) expressing various combinations of reprogramming factors. Also, GFP+ iPSCs appear earlier when *Snhg26* is overexpressed compared to control (**Fig. 7f-g** and **Supplementary Fig. 13a-f**). Second, we used a limiting dilution assay (LDA) approach to measure the frequency of reprogramming cells reaching the pluripotent state, independently from the proliferation (**Fig. 7c**). LDAs have been largely employed to measure the frequency of stem cells within a cell population (Hu *et al.*, J. of Immuno. Methods 2009; Lai *et al.*, Meth. in Cell Biol. 2022; Hollmann *et al.*, Stem Cell Res. & Ther. 2020). We showed that in the control condition, about 0.6% of cells were able to give rise to iPSCs during OKS reprogramming, whereas in the *Snhg26* overexpression, this frequency increased to about 1% (**Fig. 7h**). This result highlights that expression of *Snhg26* leads to a higher frequency of cells that reached the pluripotent state. Finally, we demonstrate in now section 7 ("SNHG26.217 lncRNA enhances the reprogramming route to pluripotency") that the increase in reprogramming efficiency is not due to the proliferation of neither the starting NPC lines (**Supplementary Fig. 12d**), nor the reprogramming cells (**Fig. 7d**) nor pluripotent stem cells (**Supplementary Fig. 12e**). Thus, these results taken together demonstrate that the apparent faster kinetics is not due to an increase in proliferation of the cells, but rather a cell state change. The effect of proliferation vs cell in change state is explained in more details in the **Supplementary Note 3**.

As for the point on an increase in cloning reprogramming, we cannot rule out that a clonal population of cells is the one increasing in reprogramming. Nonetheless, our LDA results do suggest that even if only clonal cells are reprograming then *Snhg26* does indeed accelerate their reprogramming. Otherwise there would be no difference between *Snhg26* overexpression and controls. Additionally, in human reprogramming, knock-down of *SNHG26* decreases both the number and the size of iPSC colonies positive for OCT4 staining (**Fig. 9h-i**), suggesting that reprogramming is less efficient without *SNHG26.* Altogether, these results indicate that *Snhg26* enhances the route to pluripotency without affecting cell proliferation, but rather by increasing the kinetics and/or the clonal efficiency of the process.

> *3. The role and molecular mechanism of snhg26 lncRNA in maintaining the pluripotency of embryonic stem cells.*
> *Using a knock-down strategy, the authors examined the role of the lncRNA SNHG26 in maintaining pluripotency in both murine and human embryonic stem cells (ESCs). They report that SNHG26 lncRNA Knock down reduces clonal-formation efficiency. However, this observation alone does not establish SNHG26 lncRNA as an essential factor for pluripotency maintenance in ESCs. To substantiate such a claim, the authors must perform extended serial passaging and rigorously quantify core pluripotency markers as well as lineage-differentiation genes. Otherwise, the authors should turn down their claims. In addition, Knockout of the lncRNA shows no detectable impact on core pluripotency genes in human embryonic stem cells (hESCs), raising concerns about its functional relevance.*

We agree with the reviewer that a more rigorous investigation of the importance of *SNHG26* for human pluripotency is needed. Of note, we did not perform *SNHG26* knock-out in hESCs in the first submission, but rather a knock-down (KD) in both hESCs (through CRISPRi) and during human reprogramming (using ASOs). For this re-submission, we performed RNAseq on our *SNHG26*-KD hESCs. We found that many pluripotency related genes, such as *DPPA3, UTF1, KLF4, PRDM4* and *FGF5* were down-regulated upon *SNHG26* KD (**Fig. 9c-d**), whereas the core pluripotency genes (i.e. *SOX2, NANOG, OCT4*) were not affected. This suggests that *SNHG26* may affect pluripotency but not necessarily through the regulation of its core genes. However, the fact that *Oct4* and *Nanog* genes are affected following *Snhg26* KD in mouse ESCs (**Fig. 8c**), but not in hESCs, can be explained by the fact that hESCs correspond to primed pluripotency, while mouse ESCs have a *naïve* pluripotent state (Davidson *et al.,* Development 2015; Messmer *et al.,* Cell Rep. 2019). Thus, *Snhg26* may be more important for acquisition of *naïve* pluripotency than the primed state. This is demonstrated by the expression profile of *SNHG*26 seen in **Fig. 9b** (i.e. higher in *naïve* than in primed hESCs), its expression in the early embryo (i.e. highest expressed during the genome activation phase of embryo development, **Fig. 9a** (human) and **Fig. 8b** (mouse)) and the effect of its loss on mouse blastoids formation (**Fig. 8e-h**). Moreover, during human reprogramming, *SNHG26* KD decreased both the number and the size of the iPSC colonies (**Fig. 9h-i**), while overexpression in mouse systems enhanced reprogramming, suggesting a least a conserved role in the acquisition of pluripotency. Nonetheless, we decided to tone down our claims for this current manuscript. A more detailed analysis of the role of *Snhg26* in *naïve* pluripotency is warranted as the reviewer suggested, but it is outside the scope of this manuscript, which focuses on isoform diversity of reprogramming rather than one specific gene.

<u>*MINOR POINTS*</u>

*4. In Figure 1, the design of an efficient reprogramming system is not the main research finding of this study. This section merely validates the feasibility of using long-read sequencing to detect gene expression during reprogramming.*

We agree with the reviewer that the design of our reprogramming system is not the main finding of the manuscript. We now minimized the description and characterization of the system in **Fig. 1** to reflect the reviewer's suggestion.

*5. In Figure 1, Why did the authors choose NPCs as the starting cell type for constructing the reprogramming system? In Figure 6, the expression pattern of lncRNA (2700038G22Rik) in the early stage of NPC reprogramming is significantly different from that in MEF and TTF reprogramming.*

This is an important point and not an intuitive one. We provided more details on our choice of using NPCs, which is explained in the Supplementary Note accompanying the manuscript. Briefly, we chose NPCs because they are easy to culture and transfect (Richard *et al.*, Brain Res. Mol. Brain Res. 2005; Zhang *et al.,* Stem Cell Res. Ther. 2018), they can be maintained for longer period in culture compared to MEFs (which are limited to 4-5 passages) (Parrinello *et al.*, Nat. Cell Biol. 2003). They are quickly expandable to reach the high numbers of cells needed for lrRNAseq protocols, specifically for early timepoint analysis. Also, unlike MEFs, NPCs already express *Sox2*, which facilitates and accelerates their reprogramming towards induced pluripotency (Kim *et al.*, Nature 2008). Using NPCs is thus useful when not including *c-Myc* in the cocktail of

reprogramming factors, as timelines to reprogram MEFs with OKS extend from 14 to 21 days with a decreased efficiency (Nakagawa *et al.*, Nat. Biotechnol. 2008; Sui *et al.,* Stem Cell Reports 2014; Borkent *et al.,* Stem Cell Reports 2016), whereas we obtain iPSCs as early as day 6 with NPCs (**Supplementary Note 1d**).

Regarding *2700038G22Rik* (*Snhg26*) expression pattern in early reprogramming, there is indeed an important difference between fibroblasts and NPCs, as *Snhg26* is more expressed in NPCs than MEFs or TTFs. However, its expression is first down-regulated in the early stages of NPC reprogramming, likely due to the media change (**Fig. 6d**). Indeed, our reprogramming protocol involves placing the NPCs in FBS-LIF media ("FBSL"), making the cells differentiate towards an astrocyte lineage and adopt a fibroblast-like expression profile and phenotype (**Supplementary Fig. 1a**). This leads to a decrease in *Snhg26* expression from the very beginning of reprogramming (pink bar in **Fig. 6d**). *Snhg26* then increases in expression as seen in other MEF and TTF reprogramming systems (**Fig. 6c**). **Supplementary Note 1e** and **Fig. 4a** demonstrate that our NPC reprogramming system is very similar to MEF reprogramming in terms of gene expression and alternative splicing regulations. Thus, higher expression of *2700038G22Rik* in NPCs compared to MEFs is unlikely to drastically affect the kinetics and mechanisms at play during reprogramming. Finally, knock-down of *SNHG26* during human dermal fibroblast reprogramming decreased the number and size of iPSC colonies (**Fig. 9h-i**), further suggesting that it is implicated in reprogramming, regardless of the cell source.

> *6. On page 4, lines 16–17, the statement "overactivation of c-Myc induces transcriptional and splicing events through its role in promoting proliferation" requires relevant references. Additionally, no corresponding results or explanations for this conclusion are provided in the subsequent analysis.*

We thank the reviewer for pointing this issue and apologize for missing the references in the first submission. We now added the references to the manuscript. We agree with the reviewer that we do not present results or explanations in link with this statement in our study, as the point of our study is to analyze events that are independent from *c-Myc*, as those have already been studied in the literature (Sridharan *et al.,* Cell 2009; Hirsch *et al.,* Genes and Dev. 2015; Rand *et al.,* Cell Reports 2018; Prieto *et al.,* Stem Cell Rev. and Rep. 2021). However, and as per the reviewer 3's request, we added a substantial amount of analysis delineating the difference inferred to addition or removal of *c-Myc* (OKMS vs OKS) on our results. Please refer to comments #7 and #12 of reviewer 3 to see our explanations. These results and those below show that thousands of splicing events and differentially expressed isoforms are specific to the OKMS system, so most likely provoked by *c-Myc* expression. There are approximately the same number of genes that are exclusively alternatively spliced in the OKMS (4,288 genes) or the OKS (3,799 genes) systems. However, those genes group in the same GO-terms for the distinct sets of genes. This suggests that despite using different reprogramming combinations, the cells that reprogram go through the same biological and molecular processes (in bold in the GO-terms below).

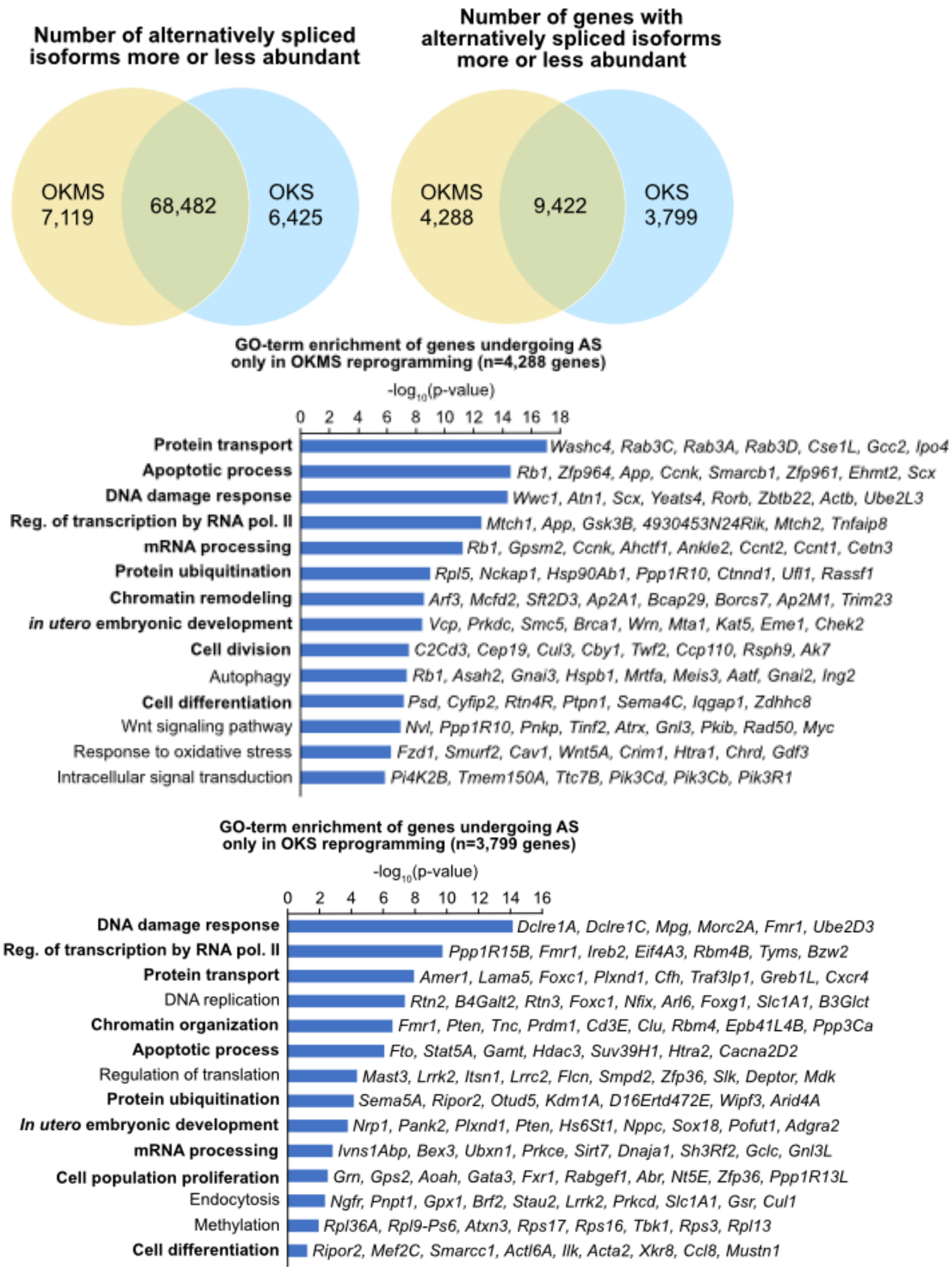


*7. In Figure 4, common splicing phenotypes of Mbnl2 and Fyn were identified by comparison with other datasets, which supports the relevant conclusions. However, the highlight of this part is "the strength of using lrRNAseq to detect new isoforms and splicing events compared to other technologies." In comparison, the analysis of the function of the novel isoforms of the 7890 gene, which is specific to lrRNAseq, can better illustrate this point.*

We thank the reviewer for highlighting this important point. We agree with the reviewer that given that we are using lrRNAseq, analyzing the functional aspects of the novel isoforms identified in our study compared to others is an advantage. Therefore, we performed functional and structural annotation of the detected isoforms (**Figures 3e**, **4j-k** and **5g**, **Supplementary Figures 7c-d** and **9i**, **Supplementary Table 2** and **Supplementary Data 4)**. For this we used the presence of

annotated PFAM as a proxy for functional isoforms. Interestingly, we detect a trend in all expressed genes, or in alternatively spliced genes which the reviewer refers to, where expression of isoforms containing PFAM domains increases during reprogramming, while their levels settle back down in ESCs (**Fig. 4j**). However, when looking at genes where isoform switching was detected, this effect is amplified and becomes significant (**Fig. 5g**). This also follows reprogramming kinetics, with the number of PFAM-annotated isoforms of the faster OKMS reprogramming peaking in the early phase, and the slower OKS reprogramming continually increasing up to the iPSC stage. Moreover, upon initiation of reprogramming, we observe a significant decrease in the percentage of non-coding isoforms from IS genes (**Fig. 5f**), which is in line with our results, where isoform switching regulates non-coding isoforms expression.

> *8. In Figure 5, it is mentioned that isoform variations of Srsf2/Srsf3 related to "mRNA processing and RNA splicing" are present. The manuscript only provides statistical and descriptive analysis of these different isoforms, and does not analyze their specific regulatory effects on RNA splicing or reprogramming. This seems to be more important.*

We thank the reviewer for this comment and agree that investigating the effect of these splicing factors on target genes is of real interest for the manuscript. As described in our response to reviewer 1, point 7: Based on our new isoform quantifications, only *Srsf2* showed significant changes in isoforms during reprogramming with both the ΔPSI events-based method and the DEIs-based method, so in this revised version we only focused on *Srsf2* and the outcome of *SRSF2* target mRNAs (identified in Han *et al.,* Mol. Cell 2017) upon reprogramming. We see that reprogramming is associated with an increase in *SRSF2* target isoforms that do not encode full-length proteins: NMD isoforms in OKMS reprogramming, and non-coding isoforms in OKS reprogramming. Interestingly, when *Srsf2*'s CDS isoforms become more prevalent in late reprogramming, we observe a concomitant decrease in NMD/non-coding isoform levels for their target mRNAs. We now provide this data in **Supplementary Fig. 9i**.

> *9. On page 14, line 6, the meaning of "meeting all the above criteria without ambiguity" is not clear. Why does the author focus on 2700038g22Rik for further analysis. What is the result of 2010204k13Rik for further analysis.*

We thank the reviewer for highlighting the need to clarify this figure, which was also asked by reviewer 1. Because the end of section 5 ("Isoform switching selectively regulates non-coding isoforms during early reprogramming") focuses on investigating IS in lncRNAs, and not necessarily protein-coding genes, we removed from **Fig. 6a** any information regarding other gene biotypes, and kept only the numbers relevant to lncRNA genes. Also, we changed the nomenclature of the two lists of genes merged in **Fig. 6a** so that it reflects better how the candidate lncRNAs were selected: the "ΔPSI events method" refers to **Fig. 3d**, and the "DEIs method" refers to **Fig. 5b**. The manuscript was updated to keep the same nomenclature as the figure and to refer to the corresponding figures. We hope this will clarify our analysis process for both the reviewer and future readers.

Regarding the selection of *2700038G22Rik* (*Snhg26*) lncRNA, as described in our response to reviewer 1, point 9, it was chosen based on integration of the expression profile of candidate lncRNAs throughout two reprogramming datasets, consistently higher expression levels in iPSCs

than in MEFs, and identification of a clear non-coding human ortholog, suggesting a possible conservation of the lncRNA, which we demonstrate in subsequent data and experiments using equivalent human systems (**Fig. 9c-i** and **Supplementary Fig. 16b-d**). We did not pursue *2010204k13Rik* further as its human ortholog appeared to be the coding gene *PAGE4*. Finally, the process of selecting *Snhg26* has now been clarified in the text and corresponding figures.

> *10. In Figure 7 or extended data figure 7, the cell morphology and colony formation under the same visual field at different time points during OKS+ Snhg26 lncRNA/ OKS reprogramming should be provided to support the conclusion that Snhg26 promotes the increase of iPSC colony formation.*

We understand the reviewer's point about keeping the same visual field throughout the reprogramming to monitor the increase in iPSC colony formation following *Snhg26* overexpression. However, in the reprogramming system that we are using, cells reach a confluency where they need to be split around D10, D14 and D18 (**Supplementary Fig. 1a**). Thus, we cannot count colonies nor follow a unique visual field to follow the colonies' evolution. Nevertheless, the OKS and OKS+*Snhg26* were split with the same ratio each time, to make sure that differences in densities were preserved between the two conditions. Also, calculation of the density of GFP+ cells is based on the volume and number of events counted per microliters in order to take into account the overall density of the cells in the wells (as explained in detail in the **Supplementary Note 2**). This way, we can follow the increase in GFP+ cells density even while splitting the cells at different timepoints. We hope that this clarifies for the reviewer why we cannot image the same field and how we bypassed this issue.

> *11. On page 17, line 4, as one of the most important findings in the manuscript, "cell intrinsic mechanisms" needs to be displayed and explained in the main figure or text.*

We thank the reviewer for pointing this out and we rephrased this part to reflect cell state change (instead of cell intrinsinc mechanism). We also added a schematic in our manuscript that now illustrate this point better (**Fig. 7c**), and a more detailed explanation is included in **Supplementary Note 3**.

> *12. In Figure 8C, the phenotype of Snhg26 knockdown is not significant. Furthermore, when analyzing the expression difference between SALL4 and REX1, the p value seems to be wrong. It would be better if the p value could be displayed in the graph.*

As suggested by the reviewer, we verified and added the p-values in the graphs in **Fig. 8c** and **Supplementary Fig. 16b** to better reflect the significance of the data.

> *13. In Extended Data Figure 9C, has the specificity of the Snhg26-specific probes been validated?*

We thank the reviewer for raising this concern. The probes have been designed carefully to take into account the presence of repetitive and/or duplicated sequences to make sure that they will target only *Snhg26* lncRNA (see reviewer 3 point 13 for more details). As an additional control, the same *in situ* hybridization experiment was performed on mouse ESCs KO for *Snhg26.* The

results have been added to **Supplementary Fig. 15c** and clearly demonstrates no signal in the KO lines, validating the specificity of the probes.

> *14. Some images lack scalebars, such as those in Figure 8F, Extended Data Figure 8H and Extended Data Figure 9G, H.*

We thank the reviewer for highlighting this oversight. The proper scalebars were now added to all relevant images.

> *15. Marker selection. DPPA3 is a more robust and representative marker for human naïve pluripotency than DPPA4.*

We thank the reviewer advising a more suitable marker. In order to give a more insightful view of how gene expression is affected following *SNHG26* knock-down, we performed lrRNAseq analysis of gene expression and displayed expression of more pluripotency-related genes in **Fig. 9c-d**. *DPPA3* was indeed down-regulated upon *SNHG26* knock-down. We added this information in the manuscript.

**Reviewer #5 (Remarks to the Author):**

> *I co-reviewed this manuscript with one of the reviewers who provided the listed reports. This is part of the Nature Communications initiative to facilitate training in peer review and to provide appropriate recognition for Early Career Researchers who co-review manuscripts.*

We would like to also thank the last reviewer for their insight and input into our manuscript.

**Response to referees**

We would like to first thank the editorial office and the reviewers for their thorough review of the manuscript and for providing constructive criticism and comments to improve it. Their comments helped us generate a much-improved revised manuscript. We are confident that we have reached the level that will satisfy both the reviewers and the editor of the journal. Please find below our point-by-point responses to the reviewers' comments. Also, our changes are highlighted in red in the manuscript text.

**Reviewer #1 (Remarks to the Author):**

> *The authors have adequately addressed the issues raised in my previous report and I am happy to recommend publication of the paper in Nature Communications.*

We would like to thank again the Reviewer for their comments that helped to substantially improve our manuscript, and we are grateful for their support towards the publication of our manuscript.

**Reviewer #2 (Remarks to the Author):**

> *I wish to thank authors for their kind responses and for thoroughly answering the comments in my report and modifying their manuscript accordingly. My concerns have now been sufficiently addressed, and I believe this round of review has contributed to improve the quality of the manuscript.*
>
> *However, I remain slightly unconvinced regarding the decision to include isoforms that lack validation in one or more of the structural sources of novelty (TSS, TTS or splice junction). The way the manuscript is currently presented, I think readers might wonder why these isoforms remain included, if sufficient validation could not be achieved. Perhaps the criteria behind this decision should be openly discussed. I understand that authors are concerned with being too stringent and perhaps removing some valuable signal from their data, but I would be cautious to point out that showing similar properties to those of fully validated isoforms is sufficient evidence to preserve them in the transcriptome.*
>
> *To avoid raising doubts among readers, I suggest authors explicitly address this decision in the paper, whether it is in the corresponding section of the manuscript or later in the discussion. I do agree that using data sources and databases that do not exactly match the system in which the data was generated might limit the extent to which isoforms can be fully validated, and this could be commented as well.*
>
> *I agree that it is reassuring, however, that all examples of biologically-relevant isoforms discussed later in the manuscript have multiple sources of evidence supporting them.*
>
> *Finally, I wish to congratulate authors on their work and, especially, on their openness to criticism and willingness to incorporate the feedback from all four reviewers. It has definitely eased the review process and should not go unnoticed.*

We would like to first express our gratitude to the Reviewer for their great input and kind encouragements. We agree with the Reviewer that if strong reasoning is not provided, then it is advisable to filter out the isoforms that are not supported one way or another by the different validation methods they suggested. Thus, we removed the transcripts that lacked validation by either junction or TSS support. Those corresponded to roughly 1.2% of all transcripts (i.e. 3,390), and we refined the final isoform list to 276,608 transcripts instead of 279,998. A graph has been added as Supplementary Fig. 6a to help readers follow this additional step. All figures, tables and text have been modified to correspond to this change.

**Reviewer #3 (Remarks to the Author):**

*We have no further comments to the authors*

We would like to thank the Reviewer for their very constructive input and for approving our manuscript.

**Reviewer #4 (Remarks to the Author):**

*The revised manuscript is substantially improved. The authors have adequately addressed several major concerns, including chimera/teratoma validation of Snhg26.217-derived iPSCs, mechanistic dissection of reprogramming efficiency vs. kinetics (limiting dilution assay, density-based analysis), and the compelling blastoid rescue experiment showing isoform-specific rescue by Snhg26.217 but not .216. However, the following issues must be resolved before acceptance:*

*Major comments:*

1. *Serial passaging after Snhg26 knockdown/KO in mouse ESCs is absent. Only 5 days of CRISPRi knockdown is shown. This is insufficient to claim a role in pluripotency maintenance. Authors must provide extended passaging (≥5 passages) with pluripotency marker quantification, or explicitly reframe the claim as "acute pluripotency perturbation" rather than maintenance.*

We thank the Reviewer and agree that our claim may still be too strong based on the data from the current manuscript. We thus used the phrasing suggested by the Reviewer to address this issue.

2. *Teratoma formation for the baseline OKMS/OKS iPSC lines (Fig. 1) remains unshown. Please either add this data or justify why chimera formation alone suffices.*

The data for control OKS iPSC lines had already been added to Supplementary Fig. 14 in the previous version of the manuscript. We now added the teratoma data for control OKMS iPSCs as well in Supplementary Fig. 14.

3. *SNHG26 knockdown in hESCs still fails to significantly downregulate OCT4/NANOG. The authors' explanation (primed vs. naïve state) is reasonable but should be more*

*prominently stated as a limitation, not a resolution. The 24–42% changes in secondary markers are modest and should not be overstated.*

We thank the Reviewer for highlighting this observation and modified the text of the manuscript to tone down our claims as requested.

*Minor comments:*

4. *Add 1–2 sentences in Results justifying the choice of NPCs as the starting cell type.*

We added a sentence to justify the choice of NPCs as the starting cell type as requested by the Reviewer. This information is also supported in the Supplementary Notes.

5. *Display exact p-values directly on Supplementary Fig. 16b graph (SALL4, REX1 changes in hESCs).*

As requested by the Reviewer, the exact p-values were already displayed in the last submission; however, we did our best to make this information more apparent in this new submission.

6. *Specify in Methods how Snhg26 ISH probe specificity was validated (e.g., knockdown control).*

Again, as previously and nicely requested by the Reviewer, the knock-out *Snhg26*$^{KO}$ ESC controls were already displayed in the last submission in Supplementary Fig. 15c; however, we did our best to make this information more apparent in this new submission, and we demonstrate the specificity of the ISH probes. The methods have also been updated accordingly to make it clearer.

7. *Verify scale bars are present in all image panels (Fig. 8F; Supplementary Figs. 13, 15).*

Indeed, scale bars had been added to all image panels in the last submission. However, scale bars had not been replicated on panels taken from the same optic field to avoid overcrowding the images. To make this new version of the manuscript even clearer, and as requested by the reviewer, we added the scale bars on all panels, independently from the optic field used.

8. *For Fig. 5 (Srsf2/Srsf3 isoforms): add at least a brief overlap analysis with published SRSF2/SRSF3 splicing targets, or explicitly frame this section as hypothesis-generating.*

Again, this overlap analysis was included in the last submission. Indeed, we had added this analysis to Supplementary Fig. 9i. In order to make it clearer, we now moved it to Fig. 5j.
