# Supplementary material for: Long-read RNA sequencing identifies non-coding isoform switching as a regulator of cell fate
Source: Nat Commun. 2026 Sep 24;17:11111. doi: 10.1038/s41467-026-78011-z (PMC13696733; doi:10.1038/s41467-026-78011-z)
Supplement: Supplementary file 4 [file 41467_2026_78011_MOESM2_ESM.pdf]

Description of Additional supplementary files

Supplementary Data 1: lrRNAseq data and QC

Supplementary Data 2: Classification file and isoform counts

Supplementary Data 3: Datasets for isoform validations, mass spectrometry ESCs table, and comparison of GTFs with Bambu, StringTie and IsoQuant

Supplementary Data 4: TSS bed file

Supplementary Data 5: Repetitive sequences bed file

Supplementary Data 6: GTFs for comparisons with Bambu, StringTie and IsoQuant

Supplementary Data 7: Alternative splicing tables

Supplementary Data 8: IsoAnnot GFF3 file

Supplementary Data 9: Isoform switch tables

Supplementary Data 10: Short-read RNAseq reprogramming tables (Stadhouders *et al.*, Kim *et al.*) processed data

Supplementary Data 11: Identification of *Snhg26*

Supplementary Data12: *Snhg26* PCR fastq reads archive

Supplementary Data 13: Validation of the role of *Snhg26*
